# Supplementary material for: Chromatin Accessibility Impacts Transcriptional Reprogramming in Oocytes
Source: Cell Rep. 2018 Jul 11;24(2):304–11. doi: 10.1016/j.celrep.2018.06.030 (PMC6057489; doi:10.1016/j.celrep.2018.06.030)
Supplement: Document S1. Supplemental Experimental Procedures and Figures S1–S4 [file mmc1.pdf]

Cell Reports, Volume *24*

## Supplemental Information

# Chromatin Accessibility Impacts Transcriptional Reprogramming in Oocytes

**Kei Miyamoto, Khoi T. Nguyen, George E. Allen, Jerome Jullien, Dinesh Kumar, Tomoki Otani, Charles R. Bradshaw, Frederick J. Livesey, Manolis Kellis, and John B. Gurdon**

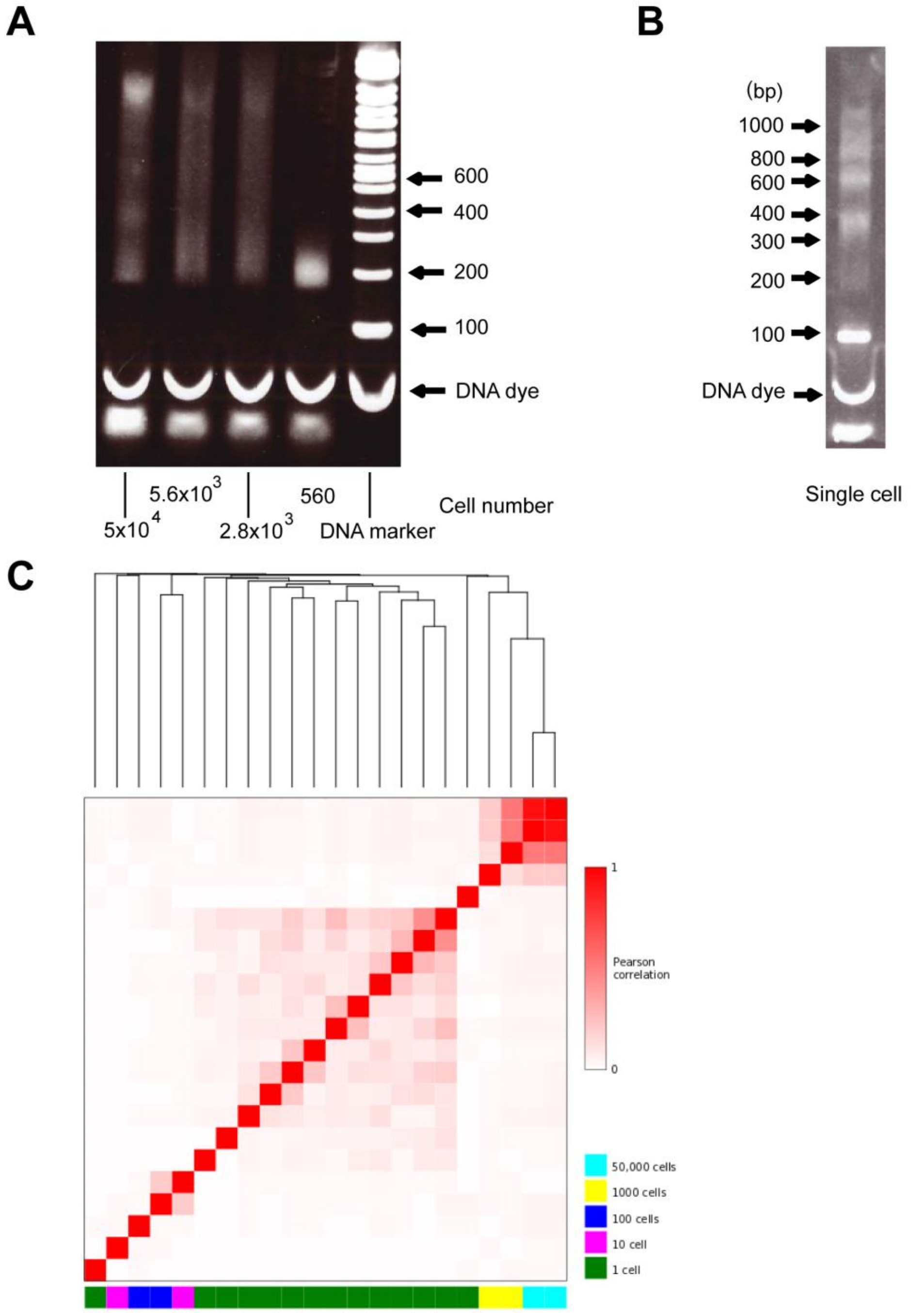


**Figure S1. ATAC-seq libraries can be produced from even a single cell, but those from the small cell numbers capture only part of open chromatin regions, related to Figure 1.**

(A) ATAC-seq libraries produced using non-diluted transposon for cutting and tagging open chromatin regions. Amplification was only seen from mono nucleosomes when 560 cells were used.

(B) An ATAC-seq library from a single C2C12 cell.

(C) Correlation between mapped ATAC-seq reads in C2C12 mouse myoblasts when 1, 10, 100, 1,000 and 50,000 cells were analyzed (22 samples). Intensity of red color denotes strong correlation (Pearson). A minimum of two replicates were performed for each group of cell numbers.

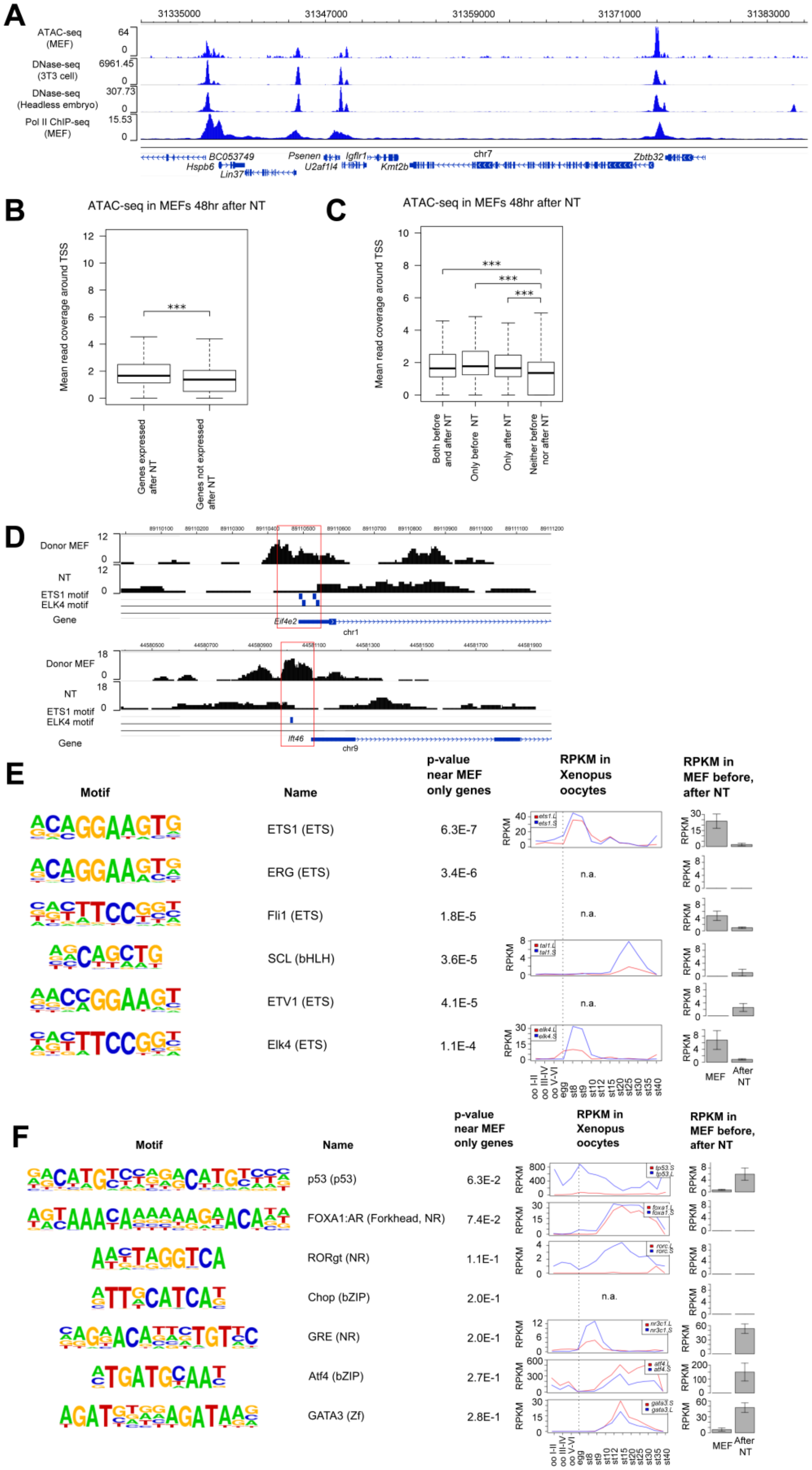


**Figure S2. Open chromatin in MEF nuclei transplanted into *Xenopus* oocytes, related to Figure 2.**

(A) Track images of ATAC-seq using 3,000 MEFs, DNase-seq using 3T3 mouse embryonic fibroblasts (GSM1003831) and Day11.5 headless embryo (GSM1014172), and ChIP-seq analyses for RNA polymerase II using MEFs (GSM918761). The same genomic region is shown for all assays.

(B) ATAC-seq reads in MEFs 48 hours after NT were compared around TSSs. Genes were divided into two categories: expressed in NT oocytes and not expressed in NT oocytes. The y-axis represents the mean read coverage in a 1 kb window centered on the TSS. *** indicates p-value < 1e-6 by the Mann-Whitney U-test.

(C) ATAC-seq reads around TSSs in MEFs 48 hours after NT were compared among different gene categories; genes expressed both before and after NT, those expressed only before NT, those expressed only after NT, and those expressed at neither timepoint. The y-axis represents the mean read coverage in a 1 kb window centered on the TSS. *** indicates p-value < 1e-6 by the Mann-Whitney U-test.

(D) ATAC-seq reads for MEFs and MEFs 48 hours after NT around the genes *Eif4e2* and *Ift46*, which were identified as genes whose expression is downregulated after NT. Locations of ETS1 and ELK4 motifs are also shown, which are motifs associated with MEF peaks near downregulated genes. Red parts indicate open chromatin regions that disappeared after NT.

(E) Sequence motifs enriched in open chromatin regions in donor MEFs near genes downregulated after NT (overlapping or within 1 kb, relative to a background of NT peaks near downregulated genes). P-values are calculated by comparing the number of foreground and background sequences with each motif using the binomial test. Expression levels of the identified transcription factors during *Xenopus* oogenesis and embryonic development are shown as line graphs (Session et al., 2016). The dotted black line separates oogenesis and embryogenesis. In case the corresponding genes are not found in *Xenopus*, no graphs are shown (n.a.). RPKM values of the identified TFs in MEFs before and after NT to *Xenopus* oocytes are also shown as bar graphs, with error bars representing ± SEM (Jullien et al., 2014).

(F) Sequence motifs enriched in open chromatin regions in NT oocytes near genes downregulated after NT (overlapping or within 1 kb, relative to a background of MEF peaks near downregulated genes).

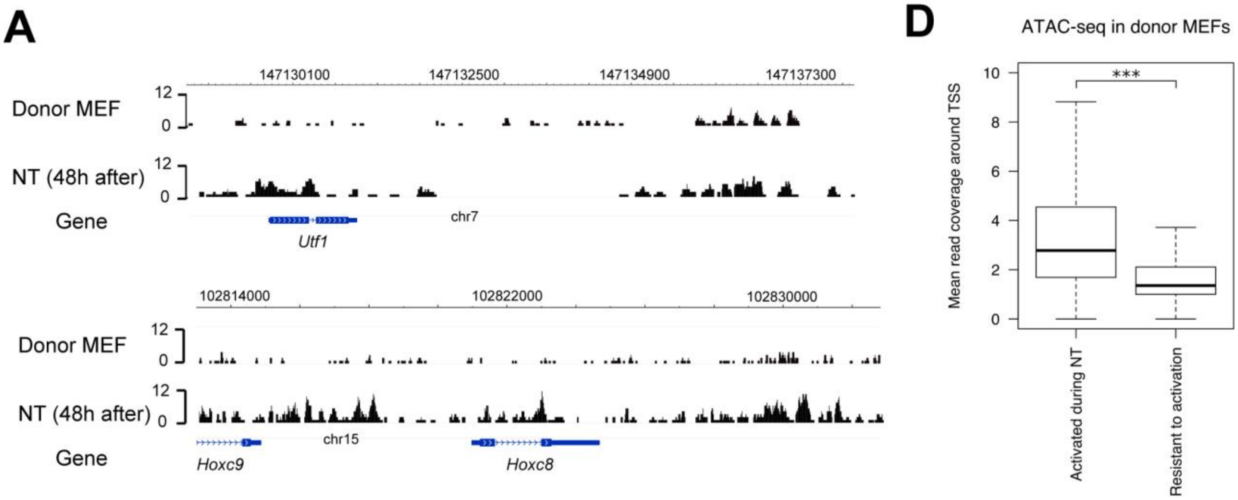


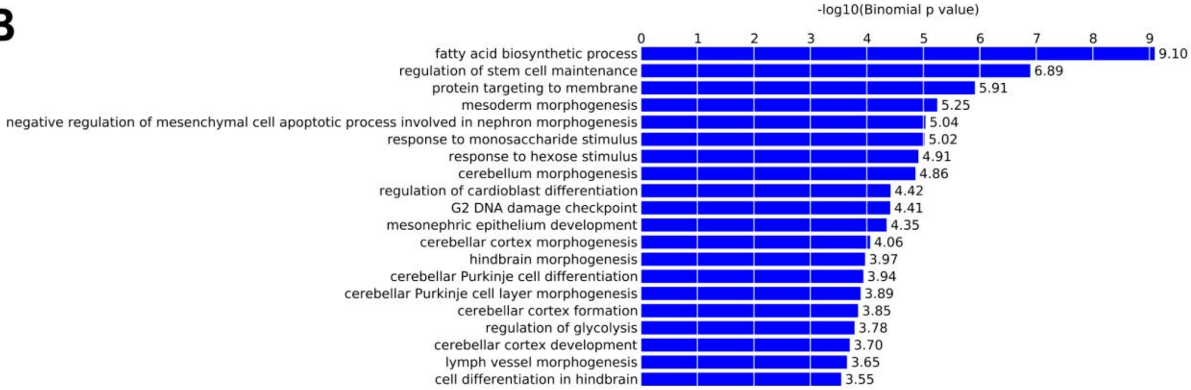


**C**

| Rank | Motif | Name | p-value | q-value |
|---|---|---|---|---|
| 1 | 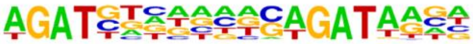 | GATA3 | 1.00E-05 | 0.0013 |
| 2 | 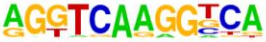 | RARG | 1.00E-05 | 0.0013 |
| 3 | 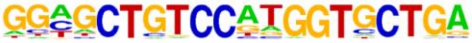 | REST-NRSF | 1.00E-02 | 0.587 |

**Figure S3. Newly produced open chromatin during reprogramming in *Xenopus* oocytes, related to Figure 3.**

(A) ATAC-seq reads for donor MEFs and MEFs 48 hours after NT.

(B) Gene categories associated with newly opened chromatin peaks after NT are identified by the Genomic Regions Enrichment of Annotations Tool (GREAT). Shown are the top 20 Gene Ontology Biological Process terms that are significant by both the region-based binomial test and the gene-based hypergeometric test and have at least a 2-fold region-based enrichment, ranked by region-based binomial p-value.

(C) Sequence motifs enriched in open chromatin regions after NT, relative to open chromatin regions in donor MEFs. Transcription factors that recognize the indicated motifs are also shown. P-values are calculated by comparing the number of foreground and background sequences with each motif using the binomial test.

(D) ATAC-seq reads around TSSs in donor MEFs were compared among different gene categories; genes expressed after NT and the MEF reprogramming-resistant genes (Jullien et al., 2017). The y-axis represents the mean read coverage in a 1 kb window centered on the TSS. *** indicates p-value < 1e-6 by the Mann-Whitney U-test.

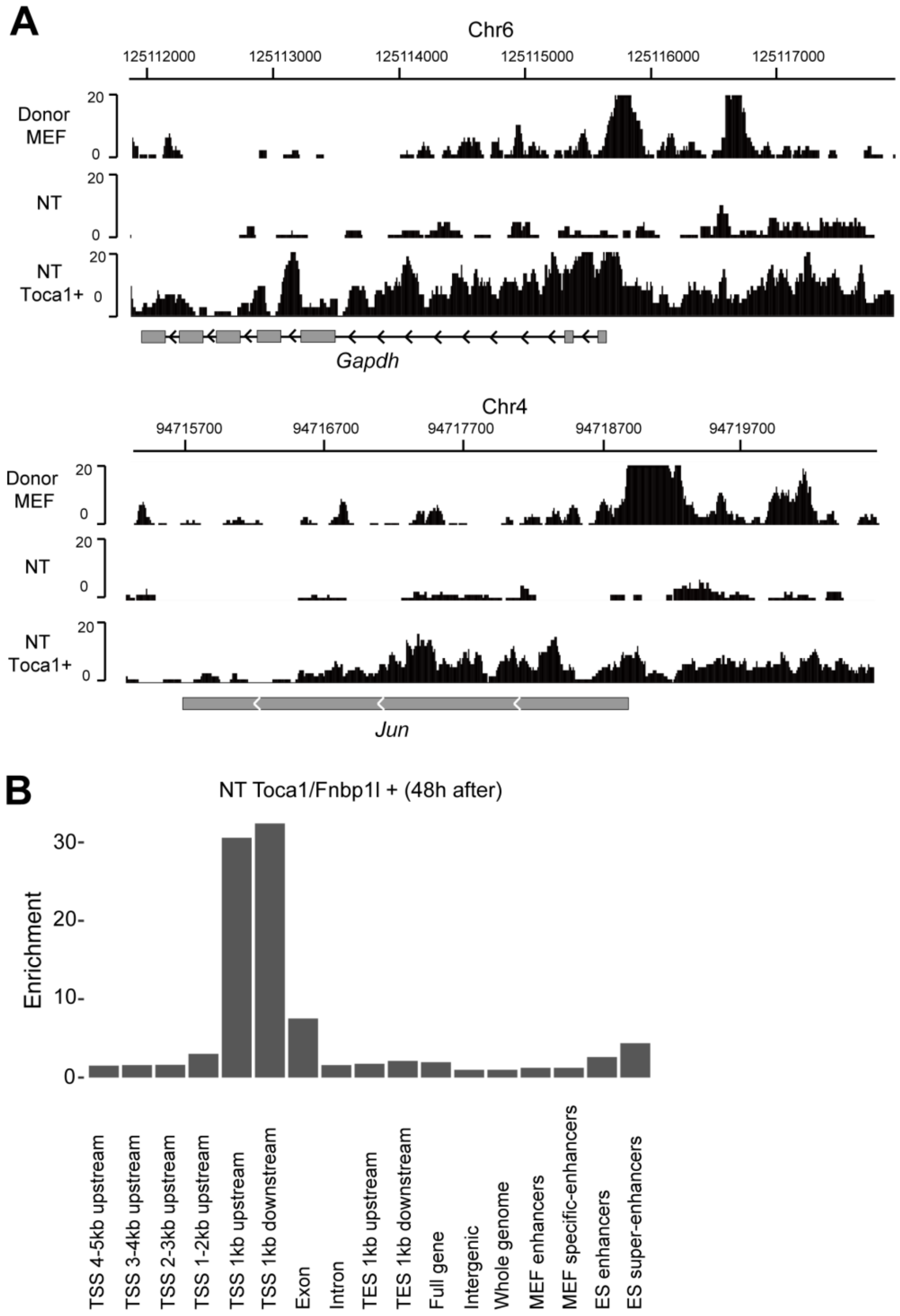


**Figure S4. Toca1/Fnbp1l overexpression in NT oocytes results in the accessible chromatin state, related to Figure 4.**

(A) ATAC-seq reads for donor MEFs, control NT oocytes, and NT oocytes overexpressed with Toca1/Fnbp1l around the *Gapdh* and *Jun* gene loci.

(B) The genomic distribution of ATAC-seq peaks detected in Toca1/Fnbp1l-overexpreessed NT oocytes. The y-axis represents the enrichment of peaks in each type of genomic region relative to the whole genome.

## SUPPLEMENTAL EXPERIMENTAL PROCEDURES

### Cell culture

MEFs were derived from E13.5 embryos hemizygous for the X-GFP transgenic allele as described previously (Jullien et al., 2014). MEFs and C2C12 myoblast cells were cultured in DMEM containing 10% FBS, penicillin, and streptomycin.

### *Xenopus laevis* oocytes and nuclear transfer

Donor MEFs were permeabilized with Streptolysin O or digitonin and approximately 300 permeabilized cells were injected into the germinal vesicle of *Xenopus* oocytes (Miyamoto et al., 2011; Miyamoto et al., 2013). Oocytes used for Figure 4A were injected with 13.8 ng of *Toca1* mRNA one day before NT (Miyamoto et al., 2011). NT oocytes were incubated at 18ºC for 48 hours. Germinal vesicles of NT oocytes were dissected in the GV isolation buffer (20 mM Tris-HCl, pH7.5, 0.5 mM $MgSO_4$, 140 mM KCl). Ten GVs equivalent to 3,000 injected nuclei were used for ATAC-seq. The isolated GVs were transferred to 500 µl of ice cold non-denaturing buffer and the GVs were collected by centrifugation at 500 g for 5 min. Pelleted GVs were resuspended in 100 µl of lysis buffer and incubated for 5 min on ice. After centrifugation at 500 g for 10 min, the collected GVs containing permeabilized nuclei were incubated in 10 µl of the transposon reaction mix. Thereafter, the ATAC-seq protocol, described in Experimental Procedures, was carried out. For transposon reaction, a total of 10 µl of the transposon mix was used and transposed DNA was purified using a QIAGEN MinElute kit before PCR amplification.

NT oocytes were also prepared or treated by various ways for experiments summarized in Figures 3 and 4. Details of RT-qPCR and confocal microscopy have been previously reported (Halley-Stott et al., 2010; Miyamoto et al., 2011). Briefly, NT oocytes were incubated in modified Barth's solution containing 0.1% BSA and antibiotics supplemented with 50 nM TSA for Figure 4B at 18ºC for 24 hours. For Figure 3B, oocytes were injected with *EGFP-dnRAR* mRNA (13.8 ng) two days before nuclear transfer. Six oocytes were pooled in order to detect transcription from transplanted nuclei by RT-qPCR. qPCR primers used are as follows; qAp1s3-F:TGCTACAGTCTCTTCTGGCCC, qAp1s3-R:TCCCTACAGCAGCGATTGC, qOct4-F:GAAGGGCAAAAGATCAAGTATTGAG, qOct4-R:GCCCCCCCTGGGAAAG, qUtf1-F: ACCAGCTGTCGACCCTGAAC, qUtf1-R: AAACGGTTTGGTCGAAGGAA, qGapdh-F:CATGGCCTTCCGTGTTCCT, qGapdh-R:GCGGCACGTCAGATCCA. Oocytes injected with EGFP-dnRAR (13.8 ng) and H2B-CFP (4.6 ng) were used for NT and subsequent confocal microscopy (ZEISS, LSM800).

### Bioinformatics

<u>ATAC-seq data processing and peak calling</u>

ATAC-seq data were processed as previously described (Corces et al., 2016). In brief, we mapped the ATAC-seq reads to the mm9 genome assembly using Bowtie2 (version 2.2.1) with the parameter –X 2000. Next, we filter out all reads meeting any of the following criteria: unmapped, mate unmapped, not primary alignment, multi-mapped, or duplicates marked by Picard MarkDuplicates (version 1.1). MACS2 (version 2.1) was used to call peaks on these reads, using the

parameters --nomodel --shift 75 --extsize 150 --keep-dup all, and a minimum p-value threshold of 0.01. To call higher confidence peaks, for a given cell type, we use only peaks called from the pooled data supported by both replicates. Next, we filtered out peaks landing in regions determined by the ENCODE Consortium (Consortium, 2012) to frequently have artificially high sequencing signal (blacklist regions). Finally, we selected only those peaks with an FDR value less than 0.01. An implementation of this protocol is available online (https://github.com/kundajelab/atac_dnase_pipelines).

Genomic distribution of peaks

The mm9 RefSeq Genes annotation available from the UCSC table browser was used to define all genomic features except enhancers. If a gene had multiple TSSs, all were included in the analysis. Enhancer and super-enhancer regions for embryonic stem cells and myotubes were previously defined by mapping regions of key transcription factor binding (Whyte et al., 2013). MEF enhancers are from the mouse ENCODE project (Shen et al., 2012) (http://chromosome.sdsc.edu/mouse/download.html). ATAC-seq peaks were also compared to myotube H3K4me3 ChIP-seq peaks (Asp et al., 2011). Reported enrichment is (a/b)/(c/d), where a is the number of peaks overlapping a given genomic feature, b is the number of total peaks, c is the number of regions corresponding to the feature, and d is the estimated number of discrete regions in the genome where the peaks and feature could overlap. Specifically, d is equal to (genome size)/(mean peak size + mean feature size), following the implementation in the bedtools fisher software tool. Significance of overlap between datasets was calculated using the hypergeometric test.

RNA-seq data

Lists of activated genes or silent genes after NT were obtained from Jullien et al. (2014) and reprogramming-resistant genes were from Jullien et al. (2017). RPKM values from Jullien et al. (2014) were used for Figures 2F, S2E and S2F.

ATAC-seq read coverage

Only reads remaining after all filtering steps described above are used for visualization (track images and heatmaps), and to calculate read coverage around TSSs. Plotted reads of ATAC-seq, DNaseI-seq (GSM1014172), and ChIP-seq (GSM769029, GSM918761) around TSSs were visualized as heatmaps using Seqplots (Stempor P (2014). seqplots: An interactive tool for visualizing NGS signals and sequence motif densities along genomic features using average plots and heatmaps. R package version 1.10.2, http://github.com/przemol/seqplots.).

Correlation between samples

For each sample, we counted the number of reads in a 1,000 bp window centered on each TSS. The correlation coefficient was calculated between pairs of samples (Pearson or Spearman, as noted in the text). For hierarchical clustering, we used average linkage and a distance metric of 1-correlation.

GREAT analysis

To assign regions to genes for Gene Ontology enrichment analysis, each gene is assigned a basal regulatory domain of a 5kb upstream and 1kb downstream of the TSS. The gene regulatory domain is extended in both directions to the nearest gene's basal domain but no more than 1000kb in one direction. Curated regulatory domains were also included. The whole genome was used as a background for the enrichment. These are the default settings implemented on the web interface for GREAT (version 3.0.0, http://bejerano.stanford.edu/great/public/html/).

Motif analysis

The HOMER tool (Heinz et al., 2010) (version 4.7, http://homer.ucsd.edu/) was used to calculate enrichment of known motifs. The set of known vertebrate motifs included with HOMER (264 motifs total) were tested for enrichment in our data.

**SUPPLEMENTAL REFERENCES**

Asp, P., Blum, R., Vethantham, V., Parisi, F., Micsinai, M., Cheng, J., Bowman, C., Kluger, Y., and Dynlacht, B.D. (2011). Genome-wide remodeling of the epigenetic landscape during myogenic differentiation. Proc Natl Acad Sci USA *108*, E149-158.

Consortium, E.P. (2012). An integrated encyclopedia of DNA elements in the human genome. Nature *489*, 57-74.

Corces, M.R., Buenrostro, J.D., Wu, B., Greenside, P.G., Chan, S.M., Koenig, J.L., Snyder, M.P., Pritchard, J.K., Kundaje, A., Greenleaf, W.J., *et al.* (2016). Lineage-specific and single-cell chromatin accessibility charts human hematopoiesis and leukemia evolution. Nat Genet *48*, 1193-1203.

Halley-Stott, R.P., Pasque, V., Astrand, C., Miyamoto, K., Simeoni, I., Jullien, J., and Gurdon, J.B. (2010). Mammalian nuclear transplantation to Germinal Vesicle stage Xenopus oocytes - a method for quantitative transcriptional reprogramming. Methods *51*, 56-65.

Heinz, S., Benner, C., Spann, N., Bertolino, E., Lin, Y.C., Laslo, P., Cheng, J.X., Murre, C., Singh, H., and Glass, C.K. (2010). Simple combinations of lineage-determining transcription factors prime cis-regulatory elements required for macrophage and B cell identities. Mol Cell *38*, 576-589.

Session, A.M., Uno, Y., Kwon, T., Chapman, J.A., Toyoda, A., Takahashi, S., Fukui, A., Hikosaka, A., Suzuki, A., Kondo, M., *et al.* (2016). Genome evolution in the allotetraploid frog Xenopus laevis. Nature *538*, 336-343.

Shen, Y., Yue, F., McCleary, D.F., Ye, Z., Edsall, L., Kuan, S., Wagner, U., Dixon, J., Lee, L., Lobanenkov, V.V., *et al.* (2012). A map of the cis-regulatory sequences in the mouse genome. Nature *488*, 116-120.

Whyte, W.A., Orlando, D.A., Hnisz, D., Abraham, B.J., Lin, C.Y., Kagey, M.H., Rahl, P.B., Lee, T.I., and Young, R.A. (2013). Master transcription factors and mediator establish super-enhancers at key cell identity genes. Cell *153*, 307-319.
