# Supplementary material for: Chromatin Accessibility Impacts Transcriptional Reprogramming in Oocytes
Source: Cell Rep. 2018 Jul 11;24(2):304–11. doi: 10.1016/j.celrep.2018.06.030 (PMC6057489; doi:10.1016/j.celrep.2018.06.030)
Supplement: Document S2. Article plus Supplemental Information [file mmc4.pdf]

Report

Cell Reports

Chromatin Accessibility Impacts Transcriptional Reprogramming in Oocytes

Graphical Abstract

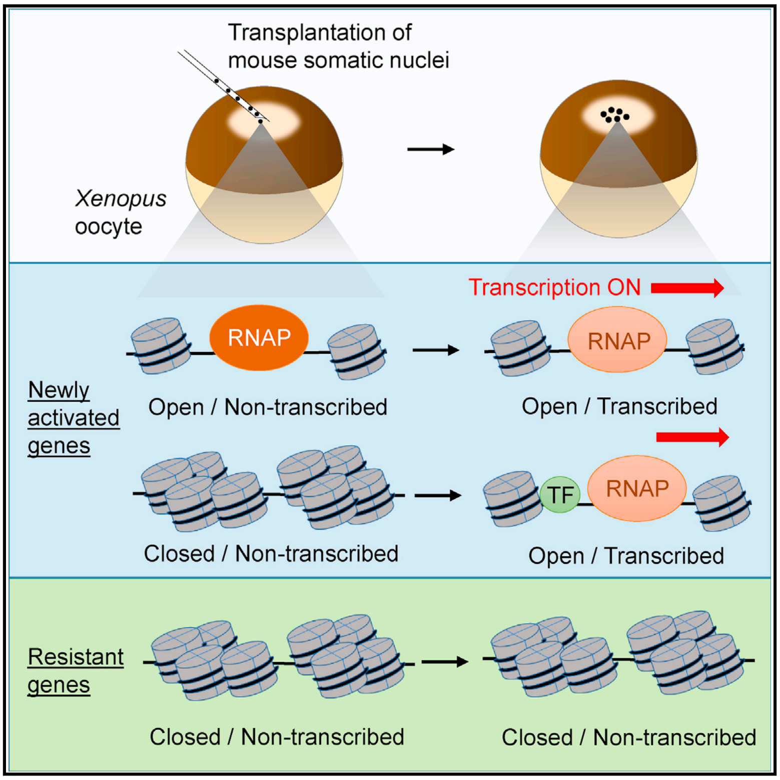

Highlights

- ATAC-seq reveals chromatin accessibility changes during reprogramming in oocytes
- Genes with open promoters are preferentially activated during reprogramming
- Transcription factors play a role in transcriptional reprogramming in oocytes
- Closed chromatin is associated with reprogramming-resistant genes

Authors

Kei Miyamoto, Khoi T. Nguyen, George E. Allen, ..., Frederick J. Livesey, Manolis Kellis, John B. Gurdon

Correspondence

kmiyamo@waka.kindai.ac.jp (K.M.), manoli@mit.edu (M.K.), j.gurdon@gurdon.cam.ac.uk (J.B.G.)

In Brief

Miyamoto et al. show genome-wide changes in chromatin accessibility during transcriptional reprogramming in oocytes using the frog nuclear transfer system. They demonstrate that donor cell chromatin states affect transcriptional reprogramming and changes in open chromatin during reprogramming are associated with specific transcription factors.

Data and Software Availability

GSE98776

Miyamoto et al., 2018, Cell Reports *24*, 304–311
July 10, 2018 © 2018 The Authors.
https://doi.org/10.1016/j.celrep.2018.06.030

CellPress

OPEN ACCESS
CellPress

Cell Reports
Report

Chromatin Accessibility Impacts Transcriptional Reprogramming in Oocytes

Kei Miyamoto,[1,2,6,*] Khoi T. Nguyen,[3,4,5] George E. Allen,[1] Jerome Jullien,[1] Dinesh Kumar,[3,4] Tomoki Otani,[1] Charles R. Bradshaw,[1] Frederick J. Livesey,[1] Manolis Kellis,[3,4,*] and John B. Gurdon[1,*]
[1]Wellcome Trust/Cancer Research UK Gurdon Institute, University of Cambridge, Cambridge CB2 1QN, United Kingdom
[2]Laboratory of Molecular Developmental Biology, Faculty of Biology-Oriented Science and Technology, Kindai University, Wakayama 649-6493, Japan
[3]Computer Science and Artificial Intelligence Laboratory, Massachusetts Institute of Technology, Cambridge, MA 02139, USA
[4]The Broad Institute of MIT and Harvard, Cambridge, MA 02142, USA
[5]Department of Biological Engineering, Massachusetts Institute of Technology, Cambridge, MA 02139, USA
[6]Lead Contact
*Correspondence: kmiyamo@waka.kindai.ac.jp (K.M.), manoli@mit.edu (M.K.), j.gurdon@gurdon.cam.ac.uk (J.B.G.)
https://doi.org/10.1016/j.celrep.2018.06.030

SUMMARY

Oocytes have a remarkable ability to reactivate silenced genes in somatic cells. However, it is not clear how the chromatin architecture of somatic cells affects this transcriptional reprogramming. Here, we investigated the relationship between the chromatin opening and transcriptional activation. We reveal changes in chromatin accessibility and their relevance to transcriptional reprogramming after transplantation of somatic nuclei into *Xenopus* oocytes. Genes that are silenced, but have pre-existing open transcription start sites in donor cells, are prone to be activated after nuclear transfer, suggesting that the chromatin signature of somatic nuclei influences transcriptional reprogramming. There are also activated genes associated with new open chromatin sites, and transcription factors in oocytes play an important role in transcriptional reprogramming from such genes. Finally, we show that genes resistant to reprogramming are associated with closed chromatin configurations. We conclude that chromatin accessibility is a central factor for successful transcriptional reprogramming in oocytes.

INTRODUCTION

Transcriptional activation is pivotal for cell fate changes and is modulated by the access of chromatin- and transcription-related factors to gene regulatory regions such as promoters and enhancers. Chromatin accessibility at gene regulatory regions affects transcriptional outcome. Pronounced nucleosome-depleted regions are found around transcription start sites (TSSs) of active genes (Teif et al., 2012). In the course of mouse embryonic development, open chromatin regions dynamically change, which is accompanied by altered transcriptional activities of the associated genes (Lu et al., 2016; Wu et al., 2016). Studying chromatin accessibility dynamics at gene regulatory regions during transcriptional activation provides insight into cell fate changes.

In order to examine chromatin accessibility, DNase sequencing (DNase-seq) has been widely used, which identifies genomic regions that can be cut by the DNaseI enzyme, known as DNaseI-hypersensitive sites (Boyle et al., 2008; Stalder et al., 1980; Thurman et al., 2012). Recently, the Assay for Transposase-Accessible Chromatin Sequencing (ATAC-seq) has been developed to examine open chromatin regions by taking advantage of the Tn5 transposon's ability to preferentially insert in open chromatin regions (Buenrostro et al., 2013). ATAC-seq is a powerful tool to map open chromatin and nucleosome positions in samples of 500–50,000 cells, producing results comparable with conventional DNase-seq but requiring orders of magnitude fewer cells (Buenrostro et al., 2013). ATAC-seq methods have also been used for single cells (Buenrostro et al., 2015; Cusanovich et al., 2015).

Using the genome-wide approaches, dynamic changes in chromatin accessibility have been investigated when transcription patterns of somatic cells are reprogrammed in induced pluripotent stem (iPS) cells. In the course of reprogramming toward iPS cells, nucleosome occupancy and open chromatin regions are changed in regulatory regions, especially at the binding sites for reprogramming transcription factors (TFs) (Li et al., 2017; West et al., 2014). The binding of reprogramming TFs to chromatin is inhibited by a closed chromatin configuration marked by histone H3 lysine 9 trimethylation (H3K9me3) (Soufi et al., 2012). Furthermore, enhancing chromatin accessibility during reprogramming by knocking down the histone chaperone CAF-1 accelerates binding of TFs and the transcriptional activation of pluripotency genes (Cheloufi et al., 2015). In contrast to iPS cells, chromatin accessibility dynamics during nuclear reprogramming in oocytes have not been well elucidated on a genome-wide scale. Nuclear transfer (NT) of somatic cells to oocytes allows efficient reprogramming in gene expression, which is induced by a different mechanism from iPS cells (Jullien et al., 2011). However, it is unclear to what extent oocytes can reprogram chromatin accessibility of somatic cells.

In this study, we examined open chromatin regions using ATAC-seq before and after reprogramming of mouse somatic nuclei in *Xenopus laevis* oocytes (Jullien et al., 2014; Miyamoto

 © 2018 The Authors.
This is an open access article under the CC BY license (http://creativecommons.org/licenses/by/4.0/).

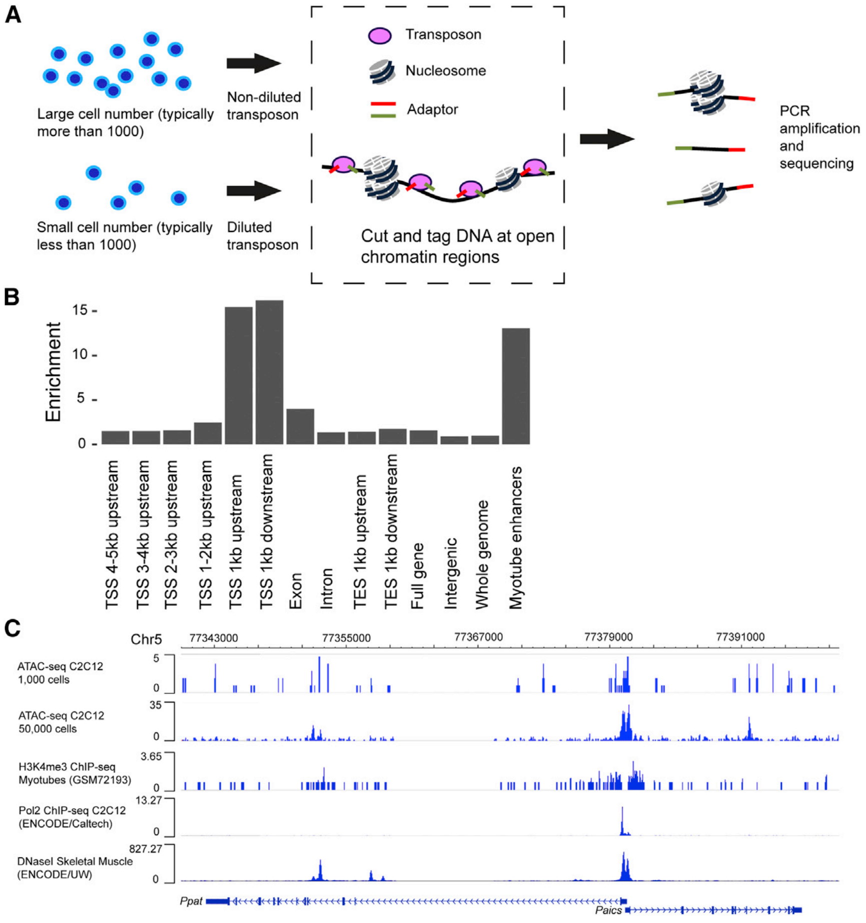

Figure 1. ATAC-Seq Enables the Identification of Open Chromatin Regions

(A) The modified ATAC-seq protocol for our experiments.

(B) The genomic distribution of ATAC-seq peaks in C2C12 mouse myoblasts, representing open chromatin. The y axis represents the enrichment of peaks in each type of genomic region relative to the whole genome. Two independently prepared ATAC-seq libraries were used for the analysis.

(C) A track image of ATAC-seq from 1,000 and 50,000 cells, chromatin immunoprecipitation sequencing (ChIP-seq) of H3K4me3 (GSM72193) and RNA polymerase II (GSM915176), and DNase-seq (GSM1014189) at the *Ppat* and *Paics* genes in mouse muscle cells. Open chromatin at the TSS is adjacent to H3K4me3 marks.

et al., 2013). This NT system enables DNA replication- and cell division-independent reprogramming of somatic nuclei so that we can assess the direct impact of oocyte factors on chromatin accessibility. We find that chromatin states of donor cells profoundly affect transcriptional reprogramming, although oocytes have an ability to open up gene regulatory regions. We show that chromatin accessibility is a key factor influencing transcriptional reprogramming in oocytes.

RESULTS AND DISCUSSION

Optimization and Evaluation of ATAC-Seq for Analyzing Cells Transplanted into Oocytes

Somatic cell nuclei transferred into oocytes have different characteristics from conventional cultured cells, in that only a small number of cells can be prepared, and those cells are difficult to permeabilize. We therefore optimized ATAC-seq protocols for our study. As reported previously (Buenrostro et al., 2013), the concentration of transposon for cutting and tagging open chromatin regions was key to a successful assay. When the cell number was small (less than 1,000 cells), dilution of the transposon prevented over-digestion of template chromatin (Figures 1A and S1A). The correct transposon concentration allowed for successful production of a DNA library with periodic nucleosome peaks even from a single cell (Figure S1B). In addition, Triton X-100 was needed to permeabilize reprogrammed cells, which contain inhibitory *Xenopus* oocyte materials only after NT.

After incorporating these modifications, we performed ATAC-seq using mouse C2C12 myoblasts from 50,000, 1,000, 100, 10, and 1 cell. When cell numbers were small (less than 100 cells), many reads were derived from mitochondria and the produced libraries showed characteristics of those with a low quality, such as duplicated reads and low library complexity. Hierarchical clustering of the mapped ATAC-seq reads around TSSs showed that the 1,000-cell and 50,000-cell samples clustered together while those created from 100, 10, and 1 cell did not cluster with the 1,000-cell and 50,000-cell samples (Figure S1C). For making use of sequencing data from the small cell numbers, multiplexing of a large number of libraries or referring to pre-existing chromatin accessibility maps would be a good way forward (Buenrostro et al., 2015; Cusanovich et al., 2015). These results suggest that ATAC-seq reads that cover the whole genome can only be obtained from libraries prepared from 1,000 or more cells, at least using our reported method. Therefore, we decided to prepare ATAC-seq libraries using more than 1,000 cells in subsequent experiments.

Genomic locations of ATAC-seq peaks, representing open chromatin sites, were examined in C2C12 cells, and 52,697 unique sites were found. Open chromatin regions were overrepresented within 1 kb of TSSs by 15.9-fold relative to the whole genome ($p < 1E{-}6$) and by 13.1-fold in myotube enhancers ($p < 1E{-}6$) (Figure 1B). Further, 40.0% of all TSSs and 40.3% of all myotube enhancers ($p < 1E{-}6$) contained ATAC-seq peaks. ATAC-seq peaks were 10.9-fold enriched in regions marked with histone H3 lysine 4 trimethylation (H3K4me3) on a genome-wide scale ($p < 1E{-}6$; Figure 1C, track image), in agreement with the previously noted association of H3K4me3 with open and active promoter elements (Guenther et al., 2007;

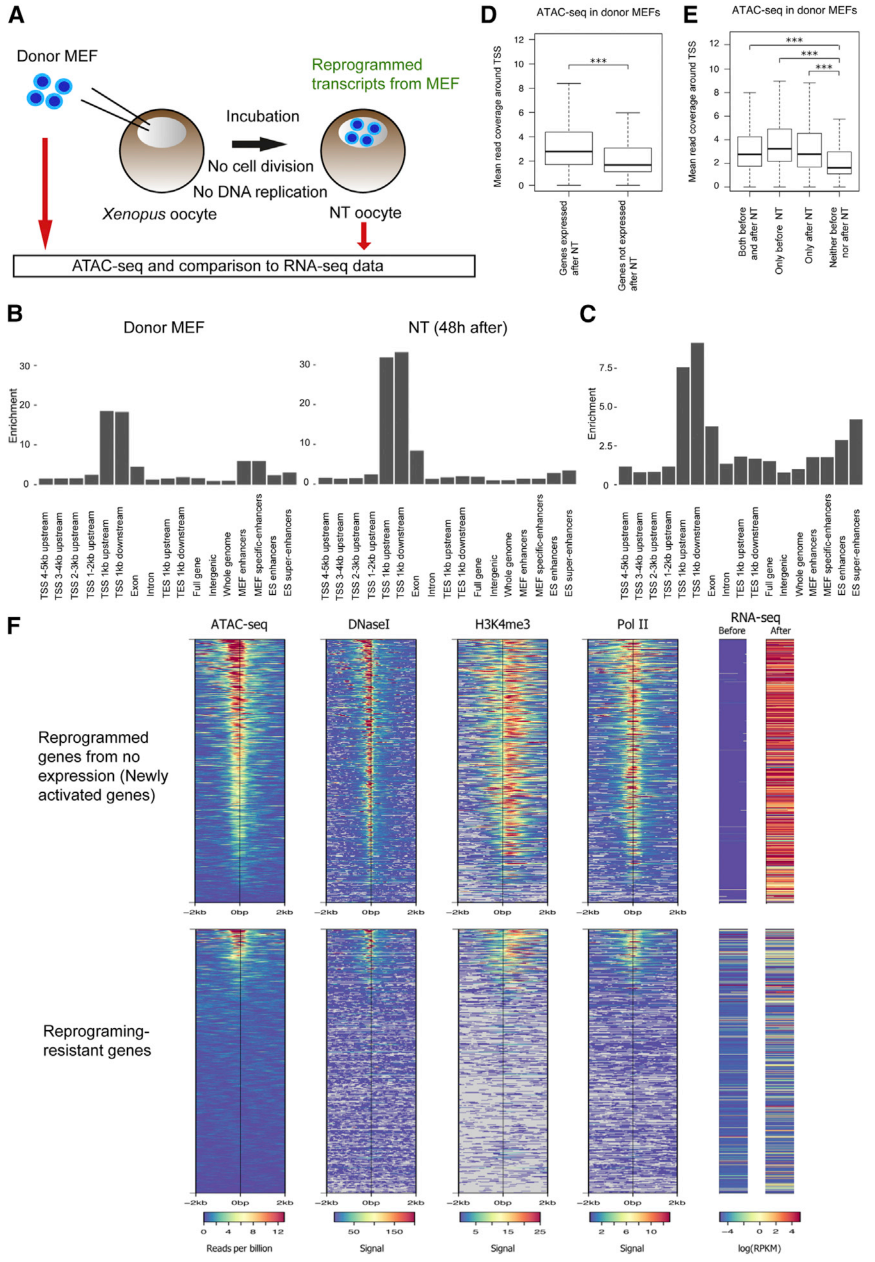

Figure 2. Genes with Open TSSs Are Preferentially Reprogrammed upon NT to *Xenopus laevis* Oocytes

(A) MEFs are transplanted into the nuclei of *Xenopus* oocytes, reprogramming their transcription. MEFs before NT and reprogrammed MEFs were used for ATAC-seq. Two biologically independent NT experiments were performed for the subsequent analyses (10 NT oocytes, equivalent to 3,000 cells, were pooled in each experiment).

(B) The genomic distribution of ATAC-seq peaks representing open chromatin before and after NT. The y axis represents the enrichment of peaks in each type of genomic region relative to the whole genome.

(C) The genomic distribution of newly appeared ATAC-seq peaks after NT.

(D) ATAC-seq reads in donor MEFs were compared around TSSs. Genes were divided into two categories: expressed in NT oocytes and not expressed in NT oocytes. The y axis represents the mean read coverage in a 1-kb window centered on the TSS.

(E) ATAC-seq reads around TSSs in donor MEFs were compared among different gene categories: genes expressed before and after NT, those expressed only before NT, those expressed only after NT, and those expressed at neither time point.

(F) Representation of signal associated with open chromatin (ATAC-seq, DNase-seq, H3K4me3 ChIP-seq, and Pol II ChIP-seq) at TSSs of MEF genes reprogrammed in *Xenopus* oocytes. RNA-seq results (Jullien et al., 2014, 2017) are shown at the right panel. Accession numbers for the DNase-seq, H3K4me3, and Pol II data are GSM1014172, GSM769029, and GSM918761, respectively.

***$p < 1E-6$ by the Mann-Whitney U test.

Heintzman et al., 2007). These data verify that our modified ATAC-seq protocol captures characteristic open chromatin features.

ATAC-Seq Reveals Changes in Chromatin Accessibility after NT of Somatic Cells to *Xenopus* Oocytes

We then examined chromatin accessibility dynamics before and after transcriptional reprogramming of somatic cells in oocytes by using the modified ATAC-seq protocol. We took advantage of the direct transcriptional reprogramming system in *Xenopus* oocytes, in which hundreds of mouse somatic nuclei transplanted into the germinal vesicle (GV), a giant nucleus of a *Xenopus* oocyte, undergo extensive transcriptional reprogramming toward an oocyte-like state within 2 days, without cell divisions and DNA replication (Figure 2A) (Jullien et al., 2014). Therefore, any changes in chromatin accessibility observed in our reprograming system are accomplished in a replication-independent manner. We performed ATAC-seq on donor mouse embryonic fibroblasts (MEFs) and on MEFs 48 hr after NT, using 3,000 cells for each sample (Figure 2A). Our ATAC-seq reads of donor MEFs resembled reads from published DNase-seq of mouse 3T3 embryonic fibroblast cells (GSM1003831, Spearman correlation with our reads = 0.84) and mouse headless embryos at day 11.5 (GSM1014172, Spearman correlation with our reads = 0.85; Figure S2A). Examination of the genomic distribution of peaks showed enrichment around TSSs both before and after NT (Figure 2B). Open chromatin peaks were also enriched at enhancers for MEFs before NT, but this enrichment was not observed after NT (Figure 2B), suggesting the closing of open chromatin at MEF-specific enhancers after NT. The relative abundance of

peaks for embryonic stem cell (ESC) enhancers did not increase much after NT, but the closing of enhancers was not observed, unlike MEF enhancers (Figure 2B, right two bars). These results suggest that MEFs, which have undergone NT, may use a different set of enhancers from those before NT to regulate gene transcription.

We next investigated genomic regions newly opened after NT. We found that 3,146 peaks appeared only after NT; those peaks present after NT were at least 5,000 bp away from a peak in MEF before NT. Such newly appeared open chromatin regions were enriched at gene regulatory regions including TSSs and embryonic stem (ES) super-enhancers (Figure 2C). The mean distance of these newly opened chromatin regions from a TSS is 61,049 bp, compared with 23,813 bp for all peaks after NT, suggesting that newly open regions are more likely to be distant from promoters. These results suggest that dynamic changes in chromatin accessibility are induced in a replication-independent manner during oocyte-mediated reprogramming.

Open Promoters of Silent Genes Are Permissive for Transcriptional Activation in Oocytes

Distribution of open chromatin peaks clearly indicates that accessible chromatin sites are mainly located at gene regulatory regions (Figures 2B and 2C). We then investigated the relationship between open chromatin at gene regulatory regions and transcriptional activation. We took advantage of our previous RNA-seq dataset in which transcriptome of donor MEFs before and after NT was revealed (Jullien et al., 2014). Mapped ATAC-seq reads were plotted around TSSs and, as expected, genes expressed after NT showed more open chromatin than non-expressed genes in these samples (Figure S2B). Intriguingly, genes expressed after NT exhibited more open chromatin than genes not expressed after NT at TSSs in the donor cells as well (Figure 2D), suggesting that pre-existing open chromatin might affect subsequent transcriptional reprogramming. To further pursue this, we examined genes newly activated after NT. These genes showed more open chromatin at TSSs than non-expressed genes in donor cells and in NT oocytes (only after NT versus neither before nor after NT; Figures 2E and S2C). Even more strikingly, a large proportion of genes activated after NT contained open chromatin already in donor cells and were also associated with H3K4me3 and unphosphorylated RNA polymerase II (Figure 2F, upper panels). This is in contrast to genes that our previous study identified as resistant to transcriptional activation in MEFs during oocyte-mediated reprogramming (Figure 2F, lower panels; reprogramming-resistant genes are discussed below) (Jullien et al., 2017). In conclusion, genes marked at their TSSs by open chromatin and other priming-associated factors such as the loading of poised RNA polymerase II (Adelman and Lis, 2012) and H3K4me3 (Voigt et al., 2013) are prone to transcriptional activation during reprogramming in oocytes. These results also imply that pre-existing open chromatin states in donor cells affect transcriptional reprogramming, further extending our previous finding that somatic cell genes abnormally maintain their expression in *Xenopus* NT embryos as somatic memory genes (Hörmanseder et al., 2017).

Transcriptional Reprogramming Is Not Solely Explained by Pre-existing Open Chromatin States at Promoters

We asked whether pre-existing open chromatin is enough to induce transcriptional reprogramming. We compared open chromatin states of downregulated genes and genes activated after reprogramming. Genes downregulated after NT (genes that were only expressed in MEFs) had a higher level of open chromatin than non-expressed genes in donor cells, as expected, but strong open chromatin states were unexpectedly maintained in downregulated genes even after NT (Figure 2E; Figure S2C, only before NT). On further inspection, we observed that, while downregulated genes retained open chromatin after NT at the global level, the open regions shifted their locations slightly (Figure S2D). In fact, some motifs were more enriched in ATAC-seq peaks detected in MEFs near these downregulated genes than in peaks detected in MEFs after NT around the same genes, and vice versa (Figures S2E and S2F). As expected, the TFs with motifs more enriched in MEF peaks tended to be more expressed in MEFs and not expressed in *Xenopus* oocytes, while the TFs with motifs more enriched in post-NT peaks tended to be more expressed in MEFs after NT and/or in *Xenopus* oocytes (Figures S2E and S2F). These results suggest that oocytes utilize different TFs from somatic cells to maintain the open chromatin near genes expressed only before NT. These regions may stay open after NT as a secondary effect of the oocyte transcriptional regulators after NT, but without somatic factors, transcription from these genes is not efficiently performed even in the presence of open chromatin. This idea is in good accordance with our previous finding that the removal of somatic transcriptional machinery and the loading of the oocyte counterpart is observed after NT to oocytes (Jullien et al., 2014).

Oocyte-Mediated Reprogramming Involves Opening of Closed Chromatin

Although many genes are transcribed from pre-existing open TSSs (Figure 2F), some genes acquire open chromatin from the closed state during oocyte reprogramming. 3,146 ATAC-seq peaks were detected only in NT samples, but not in donor MEFs (Table S1), and 497 genes without an ATAC-seq peak in MEFs gained a peak after NT, such as *Utf1* and *Hoxc8*, both of which have been shown to be reprogrammed after NT (Miyamoto et al., 2013) (Figure S3A; Table S2). We also found 1,245 ATAC-seq peaks after NT that do not overlap genes and are located at least 5 kb from a TSS or a peak before NT (Table S1). These newly formed regions of open chromatin may serve as enhancers that drive transcriptional reprogramming in this system, analogous to the enhancers that open during cellular differentiation and iPS reprogramming (Wang et al., 2011a; West et al., 2014). Newly opened regions were associated with genes related to fatty acid biosynthesis, regulation of stem cell maintenance, and protein targeting to membrane, as revealed by the Genomic Regions Enrichment of Annotations Tool (GREAT) (McLean et al., 2010) (Figure S3B). Furthermore, several motifs for binding of TFs were enriched in ATAC-seq peaks after NT relative to before NT, including GATA binding protein 3 (GATA3), retinoic acid receptor gamma (RARG), and RE1-silencing transcription factor (REST) (Figure S3C). Interestingly, REST has been shown to play a key role in NT-mediated nuclear

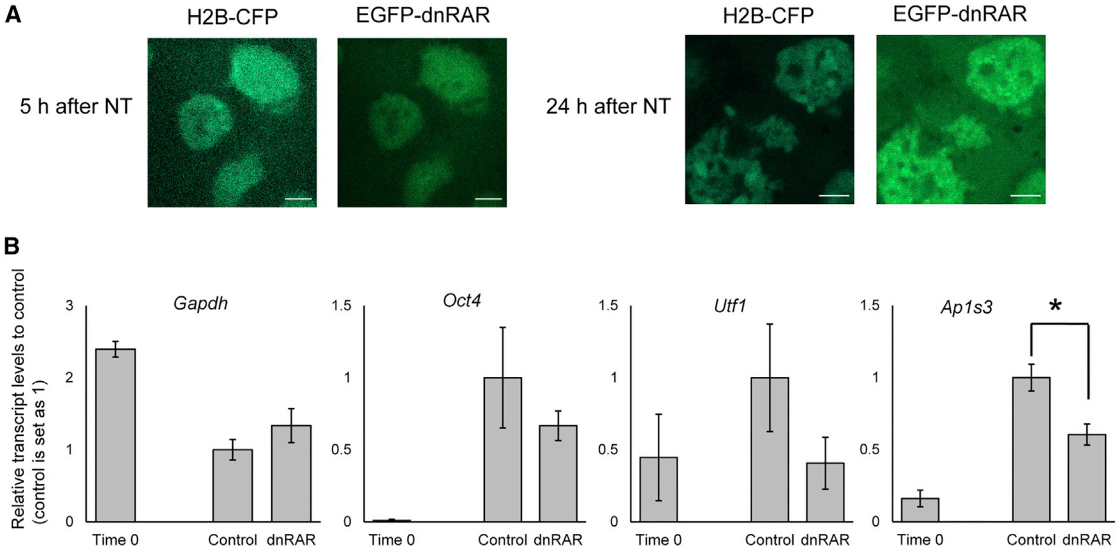

Figure 3. RAR Influences Transcriptional Reprogramming in Oocytes

(A) NT oocytes overexpressed with EGFP-dnRAR and histone H2B-CFP were subjected to confocal microscopy. EGFP-dnRAR was accumulated in the injected nuclei. Scale bars indicate 5 μm.

(B) Expression of RA-regulated genes was downregulated after overexpression of EGFP-dnRAR in NT oocytes. n = 3 (time 0) or 4 (control and dnRAR). Time 0 represents NT oocytes just after NT. Error bars represent ± SEM. *$p < 0.05$ by the Student's t test.

reprogramming in porcine oocytes (Kong et al., 2016), supporting the validity of our approach.

Transcription Factors in Oocytes Are Involved in Transcriptional Reprogramming

Because chromatin sites newly opened after NT are enriched with specific TF motifs (Figure S3C), we sought to test roles of TFs in oocytes for transcriptional reprogramming. Among the TFs identified, we focused on RAR because it showed one of the most significant hits and because RAR functions in *Xenopus* oocytes (Minucci et al., 1998). We first selected genes to test for further analyses. *Pou5f1* (known as *Oct4*) and *Utf1* have been shown to be regulated by RARs (Delacroix et al., 2010; Okazawa et al., 1991) and to be newly activated after NT to oocytes (Miyamoto et al., 2013). We also examined *Ap1s3*, which contains Retinoic Acid Response Element (RARE) at the TSS and is activated after NT. A RAR-α dominant-negative form (dnRAR) (Wang et al., 2011b) was overexpressed in NT oocytes. dnRAR was localized in transplanted MEF nuclei several hours after NT (Figure 3A). The overexpression of dnRAR in NT oocytes inhibited transcription from *Oct4*, *Utf1*, and *Ap1s3* (1.5- to 2.5-fold decrease), whereas expression of *Gapdh*, a housekeeping gene, was not affected (Figure 3B). These results suggest that newly activated genes are indeed regulated by RAR. Therefore, TFs in oocytes impact transcriptional reprogramming, possibly through opening of inaccessible sites in somatic chromatin.

Closed Chromatin Is Associated with Reprogramming-Resistant Genes

We next asked whether an opening of somatic chromatin is efficiently carried out or not after NT to oocytes. Our previous study identified genes resistant to oocyte-mediated reprogramming in MEFs (Jullien et al., 2017). These genes are not activated after nuclear transplantation of MEFs to oocytes but are transcribed after transplantation of ESCs (Jullien et al., 2017). This suggests that although transcriptional activators for the MEF reprogramming-resistant genes are present in oocytes, some feature of MEFs is preventing successful activation. Interestingly, both resistant and activated genes were transcriptionally silent in donor MEFs, but the TSSs at resistant genes in MEFs were clearly more closed compared with successfully activated genes (Figure S3D). The opening of chromatin is a central factor for successful transcriptional reprogramming, but is inefficient as is evident from the presence of the MEF reprogramming-resistant genes, suggesting that the opening of closed chromatin is a barrier for reprogramming.

Chromatin Opening Facilitates Transcriptional Reprogramming

We finally tested whether accessible chromatin states enable efficient transcriptional reprogramming. We have previously reported that enhancing nuclear actin polymerization results in efficient transcriptional reprogramming (Miyamoto et al., 2011), and that nuclear actin polymerization plays a role in chromatin decondensation (Baarlink et al., 2017). Toca1/Fnbp1l, a factor that enhances actin polymerization and transcriptional reprogramming (Miyamoto et al., 2011), was overexpressed in NT oocytes and subjected to ATAC-seq. More open chromatin peaks were found in Toca1/Fnbp1l-overexpressed NT oocytes, compared with control NT oocytes (2.6-fold increase), and 2.1-fold more genes were associated with ATAC-seq peaks by Toca1/Fnbp1l overexpression. Toca1/Fnbp1l overexpression has been shown to enhance *Oct4* activation (Miyamoto et al., 2011). Indeed, more ATAC-seq reads were found at the *Oct4*

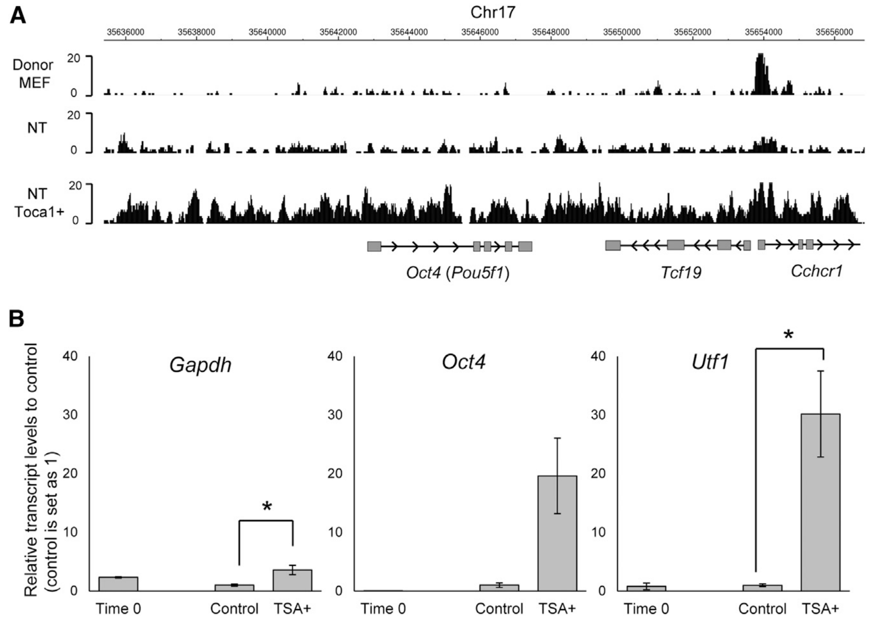

Figure 4. Efficient Transcriptional Reprogramming Is Associated with Increased Chromatin Accessibility

(A) ATAC-seq reads for donor MEFs, control NT oocytes, and NT oocytes overexpressed with Toca1/Fnbp1l around the *Oct4* gene locus.

(B) TSA treatment enhances transcriptional reprogramming. NT oocytes incubated with or without 50 nM TSA for 24 hr were used for qRT-PCR. Time 0 represents NT oocytes just after NT. n = 3 (time 0) or 4 (control and dnRAR). Error bars represent ± SEM. *$p < 0.05$ by the Student's t test.

gene locus in Toca1/Fnbp1l-overexpreessed NT oocytes than in control NT oocytes (Figure 4A), suggesting that enhanced transcriptional reprogramming is associated with the accessible chromatin state. On the other hand, genes whose transcription is not affected by Toca1/Fnbp1l overexpression (Miyamoto et al., 2011) also showed more ATAC-seq reads (Figure S4A), supporting our contention that open chromatin at TSSs is not sufficient to determine transcriptional activities. The genomic distribution of peaks found in Toca1/Fnbp1l-overexpressed NT oocytes showed enrichment around TSSs (Figure S4B), like normal NT oocytes (Figure 2B). Together, accelerated nuclear actin polymerization by Toca1/Fnbp1l overexpression seems to induce accessible chromatin globally, rather than locally, at specific loci.

We next treated NT oocytes with 50 nM Trichostatin A (TSA), a histone deacetylase inhibitor, which can induce the accessible chromatin state in somatic nuclei (Bui et al., 2010; Maalouf et al., 2009; Miyamoto et al., 2017). NT oocytes incubated with TSA increased *Oct4* and *Utf1* expression by 19.6- and 30.2-fold, respectively (Figure 4B). These results suggest that somatic chromatin is not fully opened to support transcriptional reprogramming, even after NT to oocytes, and reprogramming can be enhanced by modulating chromatin accessibility.

In summary, our study reveals genome-wide changes in chromatin accessibility in a DNA-replication-independent manner after NT of somatic cells to *Xenopus* oocytes. Transcriptional activation from the silenced genes in donor somatic cells can be accomplished from both pre-existing open chromatin and closed chromatin. In 56% of newly activated genes, pre-existing open chromatin peaks were found near TSSs before NT. These results indicate that primed genes are prone to transcriptional activation during oocyte-mediated reprogramming. Presumably, having open chromatin, H3K4me3, and poised RNA polymerase II (Pol II) at these genes helps ensure that these genes can be promptly and efficiently activated, compared with genes with closed chromatin. In contrast, only a few percentages of genes acquire obvious open chromatin sites after NT, implying that opening of closed chromatin is an inefficient process. Indeed, we found that a closed chromatin configuration at TSSs is a barrier to some genes successfully being activated during reprogramming. Chromatin opening related to transcriptional reprogramming is likely mediated by TFs such as RARs. Although oocytes possess many TFs and other machineries to relax chromatin (Jullien et al., 2011, 2014), some experimental interventions can still boost chromatin opening and transcriptional reprogramming of somatic cells. These observations suggest that somatic nuclei acquire extremely stable chromatin signatures in the course of differentiation. Increased chromatin accessibility contributes to the destabilization of somatic chromatin features and hence successful reprogramming.

EXPERIMENTAL PROCEDURES

Animals

Our work using mature *Xenopus laevis* females is covered under the UK Home Office Project License PPL 70/8591 or approved by the Animal Research Committee of Kindai University. Frog husbandry and all experiments were performed according to the relevant regulatory standards. For collecting oocytes, frogs were anesthetized by subcutaneous injection of 120 mg (in 400 μL) of Tricaine methanesulfonate (MS222). Subsequently, the frogs were slaughtered by exsanguination under anesthesia, followed by freezing for appropriate disposal.

ATAC-Seq

ATAC-seq was performed as described previously (Buenrostro et al., 2013) with some modifications. Cultured cells were harvested, washed with PBS, then transferred to cold lysis buffer (10 mM Tris-HCl [pH 7.4], 10 mM NaCl, 3 mM MgCl2, 0.1% IGEPAL, 0.5% Triton X-100). Triton X-100 was crucial for permeabilizing cells after NT. Cells in lysis buffer were collected by centrifugation and the cell pellet resuspended in the transposon reaction mix (25 μL 2× TD Buffer [FC-121-1030; Illumina], 2.5 μL Tn5 Transposase [FC-121-1030; Illumina], 22.5 μL of Nuclease Free H_2O). A total of 50 μL of the transposon reaction was used for 50,000 cells, while 1,000 or fewer cells were incubated in 5 μL of the transposon reaction. Moreover, diluted transposon (up to 1/1,000) was used when cell numbers were 100, 10, and 1. The transposition reaction was performed at 37°C for 30 min. After the reaction, transposed DNA was purified using a Qiagen MinElute kit and used for subsequent PCR amplification. DNA from 1,000 or fewer cultured cells was directly subjected to PCR amplification without kit purification. Transposed DNA was amplified by two rounds of PCR using NEB Next High Fidelity Master Mix (M0541; New England Labs) and the Customized Nextera PCR primers in 50 μL (Buenrostro et al., 2013).

The first PCR varied from 5 to 15 cycles depending on the starting cell numbers. The number of second PCR cycles was determined by qPCR using 10% of the total PCR in order to amplify the DNA in the exponential growth phase. The amplified DNA was purified using QIAGEN PCR Cleanup kit and finally resuspended in 20 μL of elution buffer. The quality of library was checked on polyacrylamide gels and quantified using the KAPA library quantification kit for Illumina sequencing platforms (KAPA Biosystems). ATAC-seq libraries were computationally processed as described in the Supplemental Experimental Procedures.

Statistical Analysis

The number of biological replicates is shown as n. In transcriptional assays by qRT-PCR, the statistical difference was calculated by the two-tailed Student's t test. Error bars were represented as the SEM. The levels of significance were set as $^*p < 0.05$. The statistical difference in mean ATAC-seq signal around groups of genes was calculated using the two-tailed Mann-Whitney U test. The statistical significance of the overlap between ATAC-seq peaks and other genomic datasets was calculated using the hypergeometric test. These statistical tests were conducted using the R software package (version 3.4). Further computational methods are described in the Supplemental Experimental Procedures.

DATA AND SOFTWARE AVAILABILITY

The accession number for the ATAC-seq data generated in this study and reported in this paper is GEO: GSE98776.

SUPPLEMENTAL INFORMATION

Supplemental Information includes Supplemental Experimental Procedures, four figures, and two tables and can be found with this article online at https://doi.org/10.1016/j.celrep.2018.06.030.

ACKNOWLEDGMENTS

We thank Drs. W.J. Greenleaf and H.Y. Chang for providing a detailed ATAC-seq protocol and Dr. P. Liu for the dnRAR construct. We also thank T. Kobayashi, Y. Tsukamoto, and C. Kimura for technical support. K.M. is supported by the Human Frontier Science Program (grant RGP0021/2016), JSPS KAKENHI (grants JP16H01321, JP16H01222, and JP17H05045), and Kindai University (research grant 17-II-3). The Gurdon laboratory is supported by grants from Wellcome Trust (101050/Z/13/Z) and the MRC (MR/K011022/1), and supported by the Gurdon Institute core grant from Cancer Research UK (C6946/A14492) and the Wellcome Trust (092096/Z/10/Z). K.T.N. was supported by grants from the NIH (5-R01-GM113708 and 5-U41-HG007000).

AUTHOR CONTRIBUTIONS

K.M. conceived the experiments and wrote the manuscript. K.M., K.T.N., and J.J. designed the experiments. K.M., T.O., and J.B.G. carried out experiments. K.T.N., D.K., G.E.A., and C.R.B. performed bioinformatics analyses. K.T.N., J.J., and J.B.G. edited the manuscript. F.J.L., M.K., and J.B.G. supervised research.

DECLARATION OF INTERESTS

The authors declare no competing interests.

Received: June 22, 2017
Revised: March 18, 2018
Accepted: June 7, 2018
Published: July 10, 2018

REFERENCES

Adelman, K., and Lis, J.T. (2012). Promoter-proximal pausing of RNA polymerase II: emerging roles in metazoans. Nat. Rev. Genet. *13*, 720–731.

Baarlink, C., Plessner, M., Sherrard, A., Morita, K., Misu, S., Virant, D., Kleinschnitz, E.M., Harniman, R., Alibhai, D., Baumeister, S., et al. (2017). A transient pool of nuclear F-actin at mitotic exit controls chromatin organization. Nat. Cell Biol. *19*, 1389–1399.

Boyle, A.P., Davis, S., Shulha, H.P., Meltzer, P., Margulies, E.H., Weng, Z., Furey, T.S., and Crawford, G.E. (2008). High-resolution mapping and characterization of open chromatin across the genome. Cell *132*, 311–322.

Buenrostro, J.D., Giresi, P.G., Zaba, L.C., Chang, H.Y., and Greenleaf, W.J. (2013). Transposition of native chromatin for fast and sensitive epigenomic profiling of open chromatin, DNA-binding proteins and nucleosome position. Nat. Methods *10*, 1213–1218.

Buenrostro, J.D., Wu, B., Litzenburger, U.M., Ruff, D., Gonzales, M.L., Snyder, M.P., Chang, H.Y., and Greenleaf, W.J. (2015). Single-cell chromatin accessibility reveals principles of regulatory variation. Nature *523*, 486–490.

Bui, H.T., Wakayama, S., Kishigami, S., Park, K.K., Kim, J.H., Thuan, N.V., and Wakayama, T. (2010). Effect of trichostatin A on chromatin remodeling, histone modifications, DNA replication, and transcriptional activity in cloned mouse embryos. Biol. Reprod. *83*, 454–463.

Cheloufi, S., Elling, U., Hopfgartner, B., Jung, Y.L., Murn, J., Ninova, M., Hubmann, M., Badeaux, A.I., Euong Ang, C., Tenen, D., et al. (2015). The histone chaperone CAF-1 safeguards somatic cell identity. Nature *528*, 218–224.

Cusanovich, D.A., Daza, R., Adey, A., Pliner, H.A., Christiansen, L., Gunderson, K.L., Steemers, F.J., Trapnell, C., and Shendure, J. (2015). Multiplex single cell profiling of chromatin accessibility by combinatorial cellular indexing. Science *348*, 910–914.

Delacroix, L., Moutier, E., Altobelli, G., Legras, S., Poch, O., Choukrallah, M.A., Bertin, I., Jost, B., and Davidson, I. (2010). Cell-specific interaction of retinoic acid receptors with target genes in mouse embryonic fibroblasts and embryonic stem cells. Mol. Cell. Biol. *30*, 231–244.

Guenther, M.G., Levine, S.S., Boyer, L.A., Jaenisch, R., and Young, R.A. (2007). A chromatin landmark and transcription initiation at most promoters in human cells. Cell *130*, 77–88.

Heintzman, N.D., Stuart, R.K., Hon, G., Fu, Y., Ching, C.W., Hawkins, R.D., Barrera, L.O., Van Calcar, S., Qu, C., Ching, K.A., et al. (2007). Distinct and predictive chromatin signatures of transcriptional promoters and enhancers in the human genome. Nat. Genet. *39*, 311–318.

Hörmanseder, E., Simeone, A., Allen, G.E., Bradshaw, C.R., Figlmüller, M., Gurdon, J., and Jullien, J. (2017). H3K4 methylation-dependent memory of somatic cell identity inhibits reprogramming and development of nuclear transfer embryos. Cell Stem Cell *21*, 135–143.e6.

Jullien, J., Pasque, V., Halley-Stott, R.P., Miyamoto, K., and Gurdon, J.B. (2011). Mechanisms of nuclear reprogramming by eggs and oocytes: a deterministic process? Nat. Rev. Mol. Cell Biol. *12*, 453–459.

Jullien, J., Miyamoto, K., Pasque, V., Allen, G.E., Bradshaw, C.R., Garrett, N.J., Halley-Stott, R.P., Kimura, H., Ohsumi, K., and Gurdon, J.B. (2014). Hierarchical molecular events driven by oocyte-specific factors lead to rapid and extensive reprogramming. Mol. Cell *55*, 524–536.

Jullien, J., Vodnala, M., Pasque, V., Oikawa, M., Miyamoto, K., Allen, G., David, S.A., Brochard, V., Wang, S., Bradshaw, C., et al. (2017). Gene resistance to transcriptional reprogramming following nuclear transfer is directly mediated by multiple chromatin-repressive pathways. Mol. Cell *65*, 873–884.e8.

Kong, Q.R., Xie, B.T., Zhang, H., Li, J.Y., Huang, T.Q., Wei, R.Y., and Liu, Z.H. (2016). RE1-silencing transcription factor (REST) is required for nuclear reprogramming by inhibiting transforming growth factor β signaling pathway. J. Biol. Chem. *291*, 27334–27342.

Li, D., Liu, J., Yang, X., Zhou, C., Guo, J., Wu, C., Qin, Y., Guo, L., He, J., Yu, S., et al. (2017). Chromatin accessibility dynamics during iPSC reprogramming. Cell Stem Cell *21*, 819–833.e6.

Lu, F., Liu, Y., Inoue, A., Suzuki, T., Zhao, K., and Zhang, Y. (2016). Establishing chromatin regulatory landscape during mouse preimplantation development. Cell *165*, 1375–1388.

Maalouf, W.E., Liu, Z., Brochard, V., Renard, J.P., Debey, P., Beaujean, N., and Zink, D. (2009). Trichostatin A treatment of cloned mouse embryos improves

constitutive heterochromatin remodeling as well as developmental potential to term. BMC Dev. Biol. *9*, 11.

McLean, C.Y., Bristor, D., Hiller, M., Clarke, S.L., Schaar, B.T., Lowe, C.B., Wenger, A.M., and Bejerano, G. (2010). GREAT improves functional interpretation of cis-regulatory regions. Nat. Biotechnol. *28*, 495–501.

Minucci, S., Wong, J., Blanco, J.C., Shi, Y.B., Wolffe, A.P., and Ozato, K. (1998). Retinoid receptor-induced alteration of the chromatin assembled on a ligand-responsive promoter in Xenopus oocytes. Mol. Endocrinol. *12*, 315–324.

Miyamoto, K., Pasque, V., Jullien, J., and Gurdon, J.B. (2011). Nuclear actin polymerization is required for transcriptional reprogramming of Oct4 by oocytes. Genes Dev. *25*, 946–958.

Miyamoto, K., Teperek, M., Yusa, K., Allen, G.E., Bradshaw, C.R., and Gurdon, J.B. (2013). Nuclear Wave1 is required for reprogramming transcription in oocytes and for normal development. Science *341*, 1002–1005.

Miyamoto, K., Tajima, Y., Yoshida, K., Oikawa, M., Azuma, R., Allen, G.E., Tsujikawa, T., Tsukaguchi, T., Bradshaw, C.R., Jullien, J., et al. (2017). Reprogramming towards totipotency is greatly facilitated by synergistic effects of small molecules. Biol. Open *6*, 415–424.

Okazawa, H., Okamoto, K., Ishino, F., Ishino-Kaneko, T., Takeda, S., Toyoda, Y., Muramatsu, M., and Hamada, H. (1991). The oct3 gene, a gene for an embryonic transcription factor, is controlled by a retinoic acid repressible enhancer. EMBO J. *10*, 2997–3005.

Soufi, A., Donahue, G., and Zaret, K.S. (2012). Facilitators and impediments of the pluripotency reprogramming factors' initial engagement with the genome. Cell *151*, 994–1004.

Stalder, J., Larsen, A., Engel, J.D., Dolan, M., Groudine, M., and Weintraub, H. (1980). Tissue-specific DNA cleavages in the globin chromatin domain introduced by DNAase I. Cell *20*, 451–460.

Teif, V.B., Vainshtein, Y., Caudron-Herger, M., Mallm, J.P., Marth, C., Höfer, T., and Rippe, K. (2012). Genome-wide nucleosome positioning during embryonic stem cell development. Nat. Struct. Mol. Biol. *19*, 1185–1192.

Thurman, R.E., Rynes, E., Humbert, R., Vierstra, J., Maurano, M.T., Haugen, E., Sheffield, N.C., Stergachis, A.B., Wang, H., Vernot, B., et al. (2012). The accessible chromatin landscape of the human genome. Nature *489*, 75–82.

Voigt, P., Tee, W.W., and Reinberg, D. (2013). A double take on bivalent promoters. Genes Dev. *27*, 1318–1338.

Wang, D., Garcia-Bassets, I., Benner, C., Li, W., Su, X., Zhou, Y., Qiu, J., Liu, W., Kaikkonen, M.U., Ohgi, K.A., et al. (2011a). Reprogramming transcription by distinct classes of enhancers functionally defined by eRNA. Nature *474*, 390–394.

Wang, W., Yang, J., Liu, H., Lu, D., Chen, X., Zenonos, Z., Campos, L.S., Rad, R., Guo, G., Zhang, S., et al. (2011b). Rapid and efficient reprogramming of somatic cells to induced pluripotent stem cells by retinoic acid receptor gamma and liver receptor homolog 1. Proc. Natl. Acad. Sci. USA *108*, 18283–18288.

West, J.A., Cook, A., Alver, B.H., Stadtfeld, M., Deaton, A.M., Hochedlinger, K., Park, P.J., Tolstorukov, M.Y., and Kingston, R.E. (2014). Nucleosomal occupancy changes locally over key regulatory regions during cell differentiation and reprogramming. Nat. Commun. *5*, 4719.

Wu, J., Huang, B., Chen, H., Yin, Q., Liu, Y., Xiang, Y., Zhang, B., Liu, B., Wang, Q., Xia, W., et al. (2016). The landscape of accessible chromatin in mammalian preimplantation embryos. Nature *534*, 652–657.

Cell Reports, Volume *24*

Supplemental Information

Chromatin Accessibility Impacts Transcriptional Reprogramming in Oocytes

Kei Miyamoto, Khoi T. Nguyen, George E. Allen, Jerome Jullien, Dinesh Kumar, Tomoki Otani, Charles R. Bradshaw, Frederick J. Livesey, Manolis Kellis, and John B. Gurdon

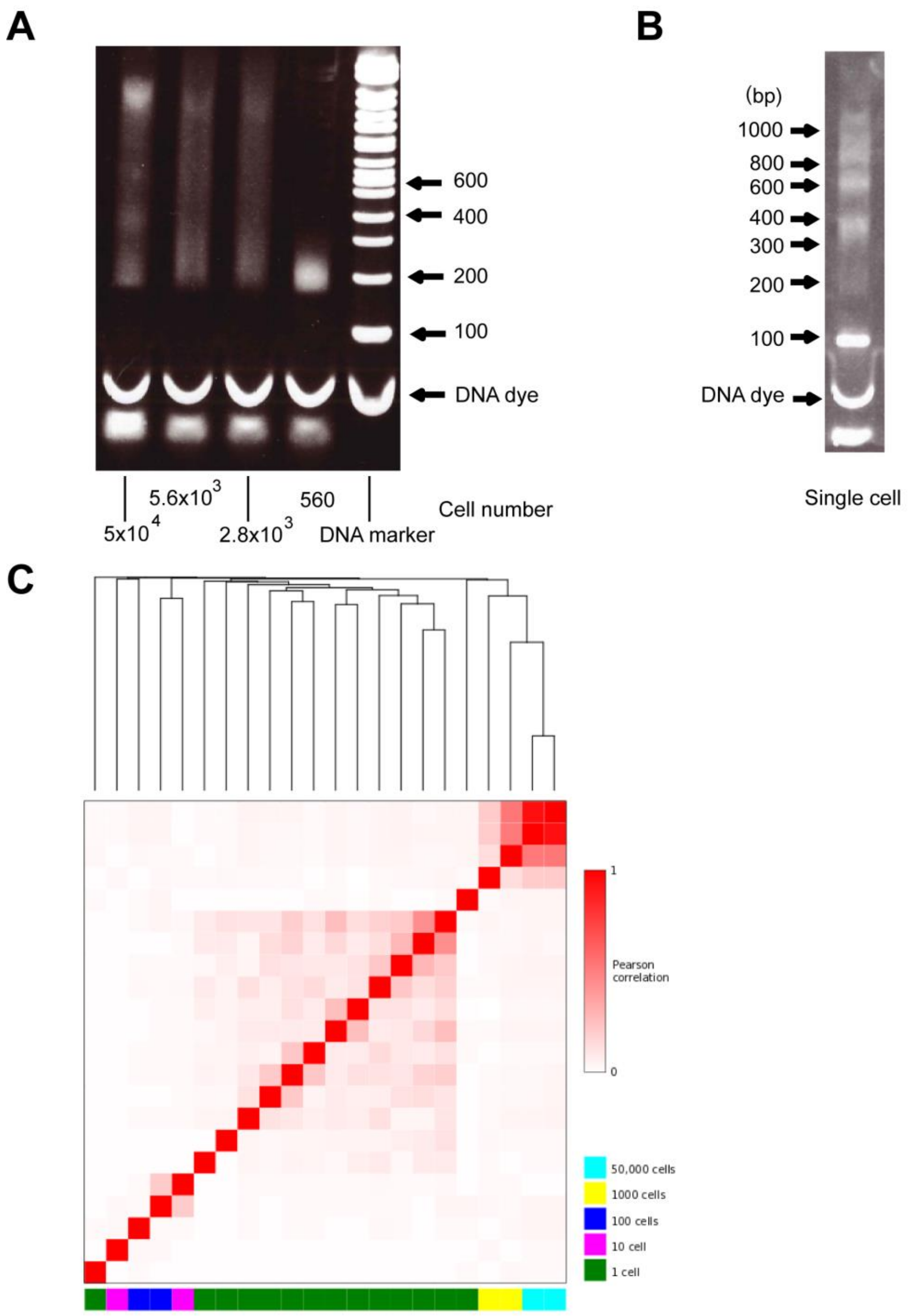

Figure S1. ATAC-seq libraries can be produced from even a single cell, but those from the small cell numbers capture only part of open chromatin regions, related to Figure 1.

(A) ATAC-seq libraries produced using non-diluted transposon for cutting and tagging open chromatin regions. Amplification was only seen from mono nucleosomes when 560 cells were used.

(B) An ATAC-seq library from a single C2C12 cell.

(C) Correlation between mapped ATAC-seq reads in C2C12 mouse myoblasts when 1, 10, 100, 1,000 and 50,000 cells were analyzed (22 samples). Intensity of red color denotes strong correlation (Pearson). A minimum of two replicates were performed for each group of cell numbers.

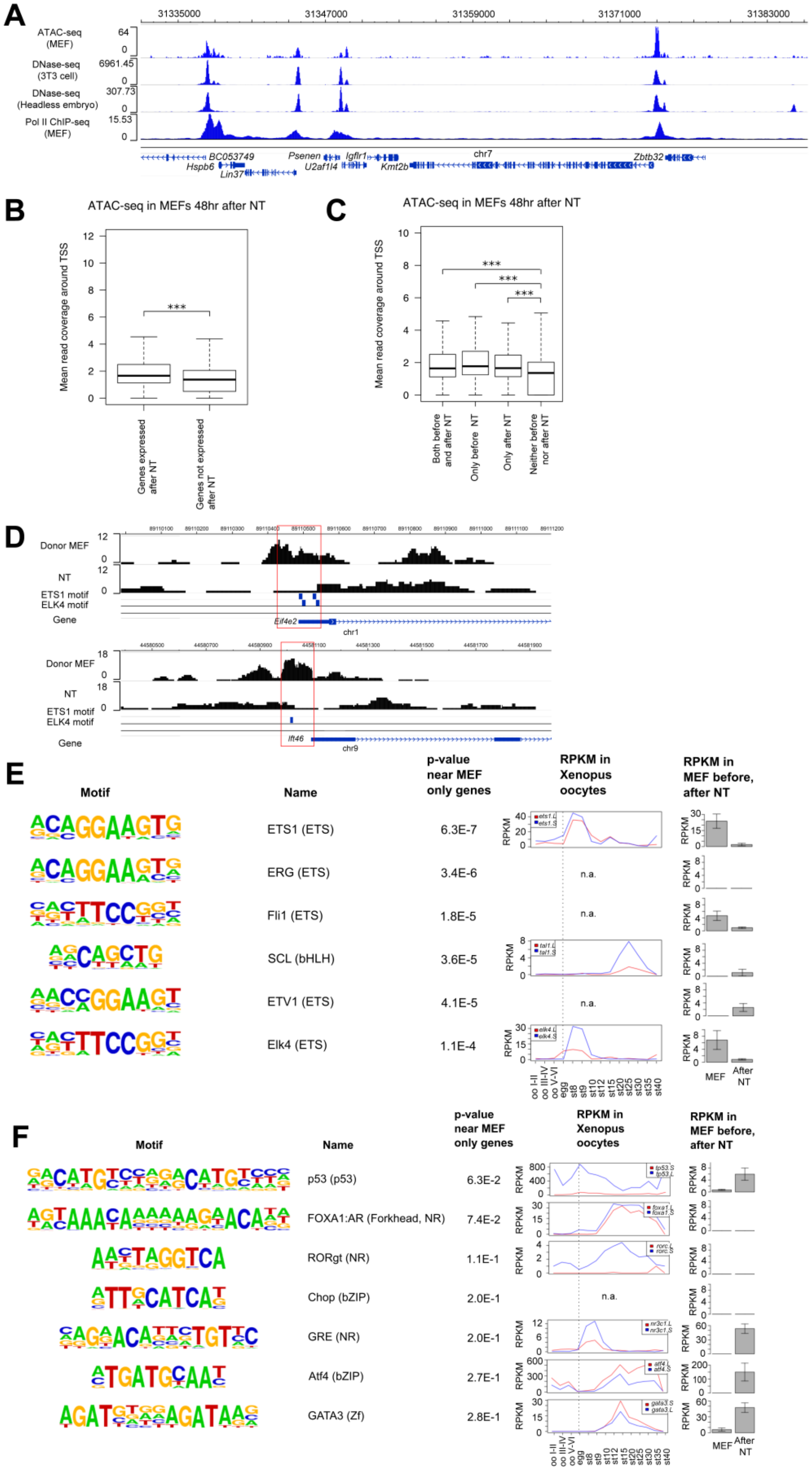

Figure S2. Open chromatin in MEF nuclei transplanted into *Xenopus* oocytes, related to Figure 2.

(A) Track images of ATAC-seq using 3,000 MEFs, DNase-seq using 3T3 mouse embryonic fibroblasts (GSM1003831) and Day11.5 headless embryo (GSM1014172), and ChIP-seq analyses for RNA polymerase II using MEFs (GSM918761). The same genomic region is shown for all assays.

(B) ATAC-seq reads in MEFs 48 hours after NT were compared around TSSs. Genes were divided into two categories: expressed in NT oocytes and not expressed in NT oocytes. The y-axis represents the mean read coverage in a 1 kb window centered on the TSS. *** indicates p-value < 1e-6 by the Mann-Whitney U-test.

(C) ATAC-seq reads around TSSs in MEFs 48 hours after NT were compared among different gene categories; genes expressed both before and after NT, those expressed only before NT, those expressed only after NT, and those expressed at neither timepoint. The y-axis represents the mean read coverage in a 1 kb window centered on the TSS. *** indicates p-value < 1e-6 by the Mann-Whitney U-test.

(D) ATAC-seq reads for MEFs and MEFs 48 hours after NT around the genes *Eif4e2* and *Ift46*, which were identified as genes whose expression is downregulated after NT. Locations of ETS1 and ELK4 motifs are also shown, which are motifs associated with MEF peaks near downregulated genes. Red parts indicate open chromatin regions that disappeared after NT.

(E) Sequence motifs enriched in open chromatin regions in donor MEFs near genes downregulated after NT (overlapping or within 1 kb, relative to a background of NT peaks near downregulated genes). P-values are calculated by comparing the number of foreground and background sequences with each motif using the binomial test. Expression levels of the identified transcription factors during *Xenopus* oogenesis and embryonic development are shown as line graphs (Session et al., 2016). The dotted black line separates oogenesis and embryogenesis. In case the corresponding genes are not found in *Xenopus*, no graphs are shown (n.a.). RPKM values of the identified TFs in MEFs before and after NT to *Xenopus* oocytes are also shown as bar graphs, with error bars representing ± SEM (Jullien et al., 2014).

(F) Sequence motifs enriched in open chromatin regions in NT oocytes near genes downregulated after NT (overlapping or within 1 kb, relative to a background of MEF peaks near downregulated genes).

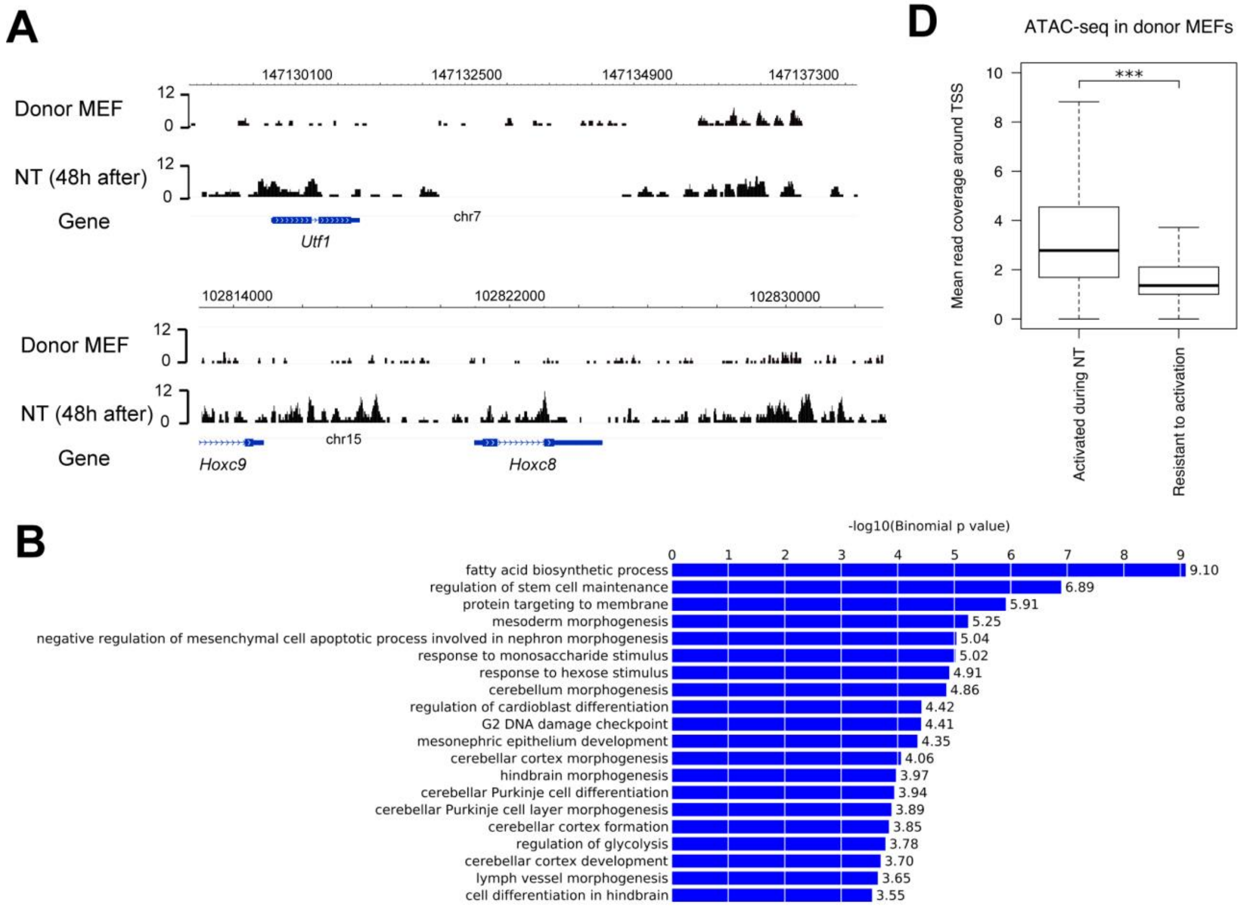

C

Rank	Motif	Name	p-value	q-value
1	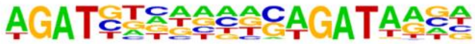	GATA3	1.00E-05	0.0013
2	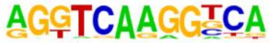	RARG	1.00E-05	0.0013
3	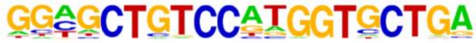	REST-NRSF	1.00E-02	0.587

Figure S3. Newly produced open chromatin during reprogramming in *Xenopus* oocytes, related to Figure 3.

(A) ATAC-seq reads for donor MEFs and MEFs 48 hours after NT.

(B) Gene categories associated with newly opened chromatin peaks after NT are identified by the Genomic Regions Enrichment of Annotations Tool (GREAT). Shown are the top 20 Gene Ontology Biological Process terms that are significant by both the region-based binomial test and the gene-based hypergeometric test and have at least a 2-fold region-based enrichment, ranked by region-based binomial p-value.

(C) Sequence motifs enriched in open chromatin regions after NT, relative to open chromatin regions in donor MEFs. Transcription factors that recognize the indicated motifs are also shown. P-values are calculated by comparing the number of foreground and background sequences with each motif using the binomial test.

(D) ATAC-seq reads around TSSs in donor MEFs were compared among different gene categories; genes expressed after NT and the MEF reprogramming-resistant genes (Jullien et al., 2017). The y-axis represents the mean read coverage in a 1 kb window centered on the TSS. *** indicates p-value < 1e-6 by the Mann-Whitney U-test.

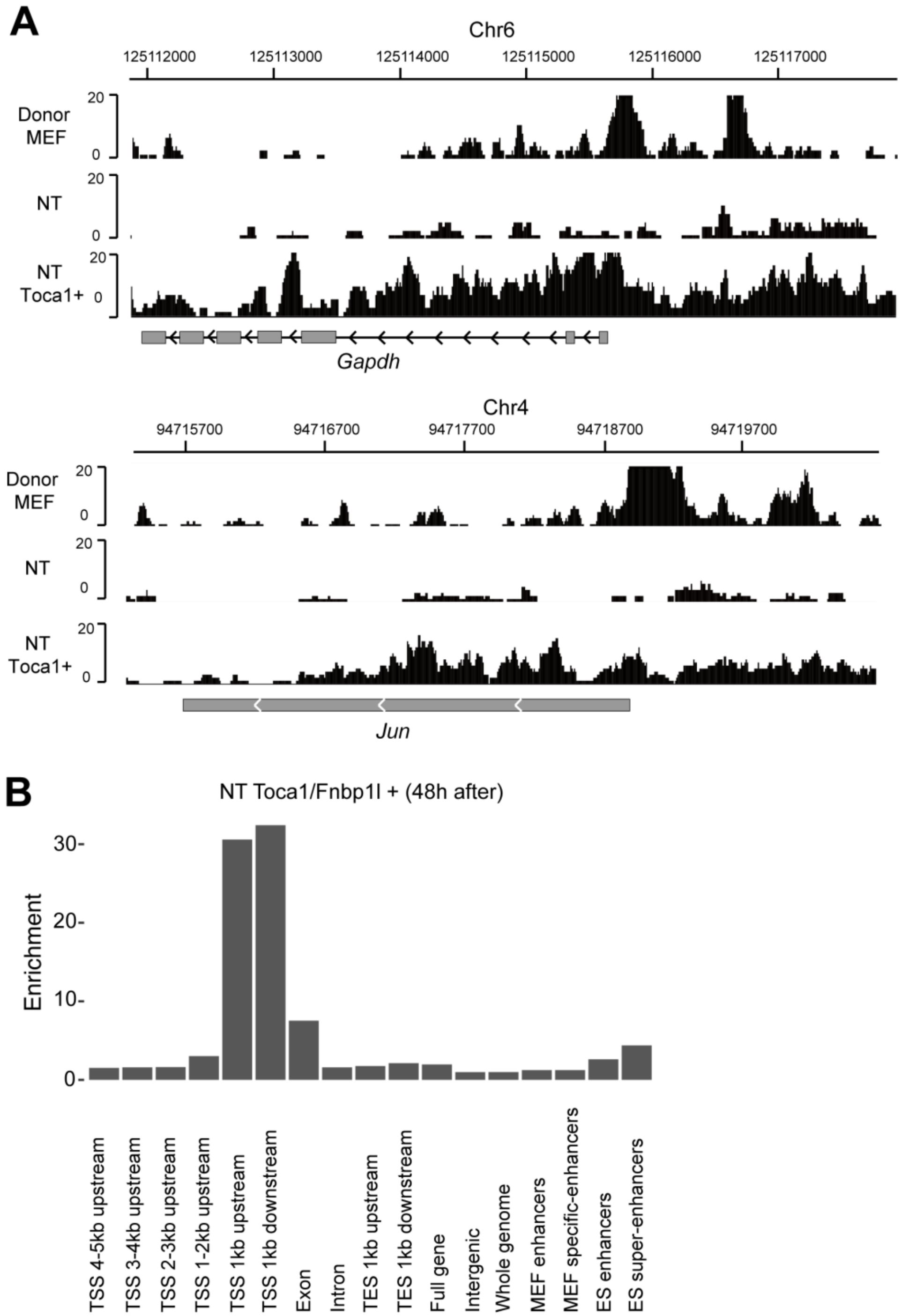

Figure S4. Toca1/Fnbp1l overexpression in NT oocytes results in the accessible chromatin state, related to Figure 4.

(A) ATAC-seq reads for donor MEFs, control NT oocytes, and NT oocytes overexpressed with Toca1/Fnbp1l around the *Gapdh* and *Jun* gene loci.

(B) The genomic distribution of ATAC-seq peaks detected in Toca1/Fnbp1l-overexpreessed NT oocytes. The y-axis represents the enrichment of peaks in each type of genomic region relative to the whole genome.

SUPPLEMENTAL EXPERIMENTAL PROCEDURES

Cell culture

MEFs were derived from E13.5 embryos hemizygous for the X-GFP transgenic allele as described previously (Jullien et al., 2014). MEFs and C2C12 myoblast cells were cultured in DMEM containing 10% FBS, penicillin, and streptomycin.

Xenopus laevis oocytes and nuclear transfer

Donor MEFs were permeabilized with Streptolysin O or digitonin and approximately 300 permeabilized cells were injected into the germinal vesicle of *Xenopus* oocytes (Miyamoto et al., 2011; Miyamoto et al., 2013). Oocytes used for Figure 4A were injected with 13.8 ng of *Toca1* mRNA one day before NT (Miyamoto et al., 2011). NT oocytes were incubated at 18ºC for 48 hours. Germinal vesicles of NT oocytes were dissected in the GV isolation buffer (20 mM Tris-HCl, pH7.5, 0.5 mM $MgSO_4$, 140 mM KCl). Ten GVs equivalent to 3,000 injected nuclei were used for ATAC-seq. The isolated GVs were transferred to 500 µl of ice cold non-denaturing buffer and the GVs were collected by centrifugation at 500 g for 5 min. Pelleted GVs were resuspended in 100 µl of lysis buffer and incubated for 5 min on ice. After centrifugation at 500 g for 10 min, the collected GVs containing permeabilized nuclei were incubated in 10 µl of the transposon reaction mix. Thereafter, the ATAC-seq protocol, described in Experimental Procedures, was carried out. For transposon reaction, a total of 10 µl of the transposon mix was used and transposed DNA was purified using a QIAGEN MinElute kit before PCR amplification.

NT oocytes were also prepared or treated by various ways for experiments summarized in Figures 3 and 4. Details of RT-qPCR and confocal microscopy have been previously reported (Halley-Stott et al., 2010; Miyamoto et al., 2011). Briefly, NT oocytes were incubated in modified Barth's solution containing 0.1% BSA and antibiotics supplemented with 50 nM TSA for Figure 4B at 18ºC for 24 hours. For Figure 3B, oocytes were injected with *EGFP-dnRAR* mRNA (13.8 ng) two days before nuclear transfer. Six oocytes were pooled in order to detect transcription from transplanted nuclei by RT-qPCR. qPCR primers used are as follows; qAp1s3-F:TGCTACAGTCTCTTCTGGCCC, qAp1s3-R:TCCCTACAGCAGCGATTGC, qOct4-F:GAAGGGCAAAAGATCAAGTATTGAG, qOct4-R:GCCCCCCCTGGGAAAG, qUtf1-F: ACCAGCTGTCGACCCTGAAC, qUtf1-R: AAACGGTTTGGTCGAAGGAA, qGapdh-F:CATGGCCTTCCGTGTTCCT, qGapdh-R:GCGGCACGTCAGATCCA. Oocytes injected with EGFP-dnRAR (13.8 ng) and H2B-CFP (4.6 ng) were used for NT and subsequent confocal microscopy (ZEISS, LSM800).

Bioinformatics

<u>ATAC-seq data processing and peak calling</u>

ATAC-seq data were processed as previously described (Corces et al., 2016). In brief, we mapped the ATAC-seq reads to the mm9 genome assembly using Bowtie2 (version 2.2.1) with the parameter –X 2000. Next, we filter out all reads meeting any of the following criteria: unmapped, mate unmapped, not primary alignment, multi-mapped, or duplicates marked by Picard MarkDuplicates (version 1.1). MACS2 (version 2.1) was used to call peaks on these reads, using the

parameters --nomodel --shift 75 --extsize 150 --keep-dup all, and a minimum p-value threshold of 0.01. To call higher confidence peaks, for a given cell type, we use only peaks called from the pooled data supported by both replicates. Next, we filtered out peaks landing in regions determined by the ENCODE Consortium (Consortium, 2012) to frequently have artificially high sequencing signal (blacklist regions). Finally, we selected only those peaks with an FDR value less than 0.01. An implementation of this protocol is available online (https://github.com/kundajelab/atac_dnase_pipelines).

Genomic distribution of peaks

The mm9 RefSeq Genes annotation available from the UCSC table browser was used to define all genomic features except enhancers. If a gene had multiple TSSs, all were included in the analysis. Enhancer and super-enhancer regions for embryonic stem cells and myotubes were previously defined by mapping regions of key transcription factor binding (Whyte et al., 2013). MEF enhancers are from the mouse ENCODE project (Shen et al., 2012) (http://chromosome.sdsc.edu/mouse/download.html). ATAC-seq peaks were also compared to myotube H3K4me3 ChIP-seq peaks (Asp et al., 2011). Reported enrichment is (a/b)/(c/d), where a is the number of peaks overlapping a given genomic feature, b is the number of total peaks, c is the number of regions corresponding to the feature, and d is the estimated number of discrete regions in the genome where the peaks and feature could overlap. Specifically, d is equal to (genome size)/(mean peak size + mean feature size), following the implementation in the bedtools fisher software tool. Significance of overlap between datasets was calculated using the hypergeometric test.

RNA-seq data

Lists of activated genes or silent genes after NT were obtained from Jullien et al. (2014) and reprogramming-resistant genes were from Jullien et al. (2017). RPKM values from Jullien et al. (2014) were used for Figures 2F, S2E and S2F.

ATAC-seq read coverage

Only reads remaining after all filtering steps described above are used for visualization (track images and heatmaps), and to calculate read coverage around TSSs. Plotted reads of ATAC-seq, DNaseI-seq (GSM1014172), and ChIP-seq (GSM769029, GSM918761) around TSSs were visualized as heatmaps using Seqplots (Stempor P (2014). seqplots: An interactive tool for visualizing NGS signals and sequence motif densities along genomic features using average plots and heatmaps. R package version 1.10.2, http://github.com/przemol/seqplots.).

Correlation between samples

For each sample, we counted the number of reads in a 1,000 bp window centered on each TSS. The correlation coefficient was calculated between pairs of samples (Pearson or Spearman, as noted in the text). For hierarchical clustering, we used average linkage and a distance metric of 1-correlation.

GREAT analysis

To assign regions to genes for Gene Ontology enrichment analysis, each gene is assigned a basal regulatory domain of a 5kb upstream and 1kb downstream of the TSS. The gene regulatory domain is extended in both directions to the nearest gene's basal domain but no more than 1000kb in one direction. Curated regulatory domains were also included. The whole genome was used as a background for the enrichment. These are the default settings implemented on the web interface for GREAT (version 3.0.0, http://bejerano.stanford.edu/great/public/html/).

Motif analysis

The HOMER tool (Heinz et al., 2010) (version 4.7, http://homer.ucsd.edu/) was used to calculate enrichment of known motifs. The set of known vertebrate motifs included with HOMER (264 motifs total) were tested for enrichment in our data.

SUPPLEMENTAL REFERENCES

Asp, P., Blum, R., Vethantham, V., Parisi, F., Micsinai, M., Cheng, J., Bowman, C., Kluger, Y., and Dynlacht, B.D. (2011). Genome-wide remodeling of the epigenetic landscape during myogenic differentiation. Proc Natl Acad Sci USA *108*, E149-158.

Consortium, E.P. (2012). An integrated encyclopedia of DNA elements in the human genome. Nature *489*, 57-74.

Corces, M.R., Buenrostro, J.D., Wu, B., Greenside, P.G., Chan, S.M., Koenig, J.L., Snyder, M.P., Pritchard, J.K., Kundaje, A., Greenleaf, W.J., *et al.* (2016). Lineage-specific and single-cell chromatin accessibility charts human hematopoiesis and leukemia evolution. Nat Genet *48*, 1193-1203.

Halley-Stott, R.P., Pasque, V., Astrand, C., Miyamoto, K., Simeoni, I., Jullien, J., and Gurdon, J.B. (2010). Mammalian nuclear transplantation to Germinal Vesicle stage Xenopus oocytes - a method for quantitative transcriptional reprogramming. Methods *51*, 56-65.

Heinz, S., Benner, C., Spann, N., Bertolino, E., Lin, Y.C., Laslo, P., Cheng, J.X., Murre, C., Singh, H., and Glass, C.K. (2010). Simple combinations of lineage-determining transcription factors prime cis-regulatory elements required for macrophage and B cell identities. Mol Cell *38*, 576-589.

Session, A.M., Uno, Y., Kwon, T., Chapman, J.A., Toyoda, A., Takahashi, S., Fukui, A., Hikosaka, A., Suzuki, A., Kondo, M., *et al.* (2016). Genome evolution in the allotetraploid frog Xenopus laevis. Nature *538*, 336-343.

Shen, Y., Yue, F., McCleary, D.F., Ye, Z., Edsall, L., Kuan, S., Wagner, U., Dixon, J., Lee, L., Lobanenkov, V.V., *et al.* (2012). A map of the cis-regulatory sequences in the mouse genome. Nature *488*, 116-120.

Whyte, W.A., Orlando, D.A., Hnisz, D., Abraham, B.J., Lin, C.Y., Kagey, M.H., Rahl, P.B., Lee, T.I., and Young, R.A. (2013). Master transcription factors and mediator establish super-enhancers at key cell identity genes. Cell *153*, 307-319.
